# Fast estimation of genetic correlation for biobank-scale data

**DOI:** 10.1016/j.ajhg.2021.11.015

**Published:** 2021-12-02

**Authors:** Yue Wu, Kathryn S. Burch, Andrea Ganna, Päivi Pajukanta, Bogdan Pasaniuc, Sriram Sankararaman

**Affiliations:** 1 Department of Computer Science, UCLA, Los Angeles, CA 90095, USA; 2 Department of Human Genetics, David Geffen School of Medicine, UCLA, Los Angeles, CA 90095, USA; 3 Bioinformatics Interdepartmental Program, UCLA, Los Angeles, CA 90095, USA; 4 Department of Pathology and Laboratory Medicine, David Geffen School of Medicine, UCLA, Los Angeles, CA 90095, USA; 5 Department of Computational Medicine, David Geffen School of Medicine, UCLA, Los Angeles, CA 90094, USA; 6 Analytic and Translational Genetics Unit, Center for Genomic Medicine, Massachusetts General Hospital, Boston, MA 02113, USA; 7 Program in Medical and Population Genetics, Broad Institute of MIT and Harvard, Cambridge, MA 02142, USA; 8 Stanley Center for Psychiatric Research, Broad Institute of MIT and Harvard, Cambridge, MA 02142, USA; 9 Institute for Precision Health, School of Medicine, UCLA, Los Angeles, CA 90095, USA

**Keywords:** genetic correlation, pleiotropy, complex traits, method of moments, biobank

## Abstract

Genetic correlation is an important parameter in efforts to understand the relationships among complex traits. Current methods that analyze individual genotype data for estimating genetic correlation are challenging to scale to large datasets. Methods that analyze summary data, while being computationally efficient, tend to yield estimates of genetic correlation with reduced precision. We propose SCORE (scalable genetic correlation estimator), a randomized method of moments estimator of genetic correlation that is both scalable and accurate. SCORE obtains more precise estimates of genetic correlations relative to summary-statistic methods that can be applied at scale; it achieves a 44% reduction in standard error relative to LD-score regression (LDSC) and a 20% reduction relative to high-definition likelihood (HDL) (averaged over all simulations). The efficiency of SCORE enables computation of genetic correlations on the UK Biobank dataset, consisting of ≈300 K individuals and ≈500 K SNPs, in a few h (orders of magnitude faster than methods that analyze individual data, such as GCTA). Across 780 pairs of traits in 291,273 unrelated white British individuals in the UK Biobank, SCORE identifies significant genetic correlation between 200 additional pairs of traits over LDSC (beyond the 245 pairs identified by both).

## Introduction

Genetic correlation is an important parameter that quantifies the genetic basis that is shared across two traits. Estimates of genetic correlation can reveal pleiotropy, uncover novel biological pathways underlying diseases, and improve the accuracy of genetic prediction.^1^

While traditionally reliant on family studies, the availability of genome-wide genetic data has led to several approaches to estimate genetic correlation from these datasets.^1^ An important class of methods for estimating genetic correlation relies on computing the restricted maximum likelihood within a bi-variate linear mixed model (LMM), termed genomic restricted maximum likelihood (GREML).^2^, ^3^, ^4^, ^5^ However, current GREML methods are computationally expensive to be applied to large-scale datasets such as the UK Biobank.^6^

While GREML methods need individual-level data, several methods,^7^, ^8^, ^9^, ^10^, ^11^, ^12^ such as linkage disequilibrium (LD)-score regression (LDSC),^7^ have been proposed for estimating genetic correlation with genome-wide association study (GWAS) summary statistics. Although methods such as LDSC often have substantially reduced computational requirements relative to GREML, LDSC estimates tend to have large standard errors that increase further when there is a mismatch between the samples used to estimate summary statistics and the reference datasets that are used to estimate LD scores.^13^ High-definition likelihood (HDL),^12^ a more recent summary-statistic-based method, has been shown to be more precise relative to LDSC. HDL, however, requires computing a singular-value decomposition (SVD) of the LD matrix, which increases its runtime. Further, recent studies^14^^,^^15^ have shown that the accuracy of genetic correlation estimates can deteriorate when there is a mismatch between reference and sample data. Thus, it is critical to develop methods for estimating genetic correlation that can work directly with large individual-level datasets.

We propose, SCORE (scalable genetic correlation estimator), a randomized method of moments (MoM) estimator of genetic correlations among traits via individual genotypes that can scale to the dataset sizes typical of the UK Biobank. While SCORE can estimate the heritability of traits as well as the genetic correlation between pairs of traits, we focus on the problem of estimating genetic correlation in this work. SCORE achieves scalability by avoiding explicit computation of the genetic relationship matrix (GRM). Instead, we show that the genetic correlation can be computed by using a *sketch* of the genotype matrix, i.e., by multiplying the genotype matrix with a small number of random vectors.

In simulations, we show that SCORE yields accurate estimates of genetic correlation across a range of genetic architectures (with varying heritability, genetic correlation, and polygenicity). Relative to summary-statistic methods that can be applied to biobank-scale data, SCORE obtains a reduction in standard error of 44% relative to LDSC and 20% relative to HDL (averaged across all simulations). Further, SCORE can estimate genetic correlation on ≈500 K SNPs in ≈300 K unrelated white British individuals in a few h, orders of magnitude faster than methods that rely on individual data (GCTA-GREML and GCTA-HE). Analyzing 780 pairs of traits in 291,273 unrelated white British individuals in the UK Biobank, the estimates of genetic correlation at 454,207 common SNPs obtained by SCORE are largely concordant with those from LDSC (Pearson correlation r=0.95). Although 245 pairs of traits are identified as having significant genetic correlation by both methods (with a Bonferroni correction for the number of pairs of traits tested), the reduced standard error of estimates from SCORE leads to the discovery of significant genetic correlations between an additional 200 pairs of traits relative to LDSC. Finally, SCORE detects a significant positive correlation between serum liver enzyme levels (alanine [ALT] and aspartate aminotransferase [AST]) and coronary-artery-disease-related traits (angina and heart attack), suggesting that coronary artery disease and liver dysfunction harbor a shared genetic component.

## Material and methods

### Bi-variate linear mixed model

We describe our model in the general setting, where the traits are not observed on the same set of individuals. Assume we have $N_{1}$ individuals for trait 1 and $N_{2}$ individuals for trait 2 of which *N* individuals $\left(N\leq N_{1},N\leq N_{2}\right)$ contain measurements for both the traits. We have defined $X_{1},X_{2}$ to be the $N_{1}\times M$ and $N_{2}\times M$ matrices of standardized genotypes obtained by centering and scaling each column of the unstandardized genotype matrices $G_{1}$ and $G_{2}$ so that $\sum_{n}x_{t,n,m}=0$ for all m∈{1,…,M},t∈{1,2}. Let $y_{1},y_{2}$ denote the two vectors of phenotypes with size $N_{1}$ and $N_{2}$, respectively. Additionally, we define an $N_{1}\times N_{2}$ indicator matrix, C, where $C_{i,j}=1$ when individual *i* among samples measured for the first phenotype and *j* in samples measured for the second phenotype refer to the same individual and 0 otherwise. We define $\beta_{1},\beta_{2}$ to be vectors of SNP effect sizes of length *M*.

We assume the following model relating a pair of traits $y_{1},y_{2}$:

(Equation 1) $$\begin{array}{l} y_{1}=X_{1}\beta_{1}+\varepsilon_{1}\\ y_{2}=X_{2}\beta_{2}+\varepsilon_{2}. \end{array}$$

For the SNP effects, we assume $E\left[\beta_{1}\right]=0,E\left[\beta_{2}\right]=0$ and

(Equation 2) $$\begin{array}{l} cov\left(\beta_{1},\beta_{1}\right)=\frac{1}{M}\sigma_{g1}^{2}I_{M}\\ cov\left(\beta_{2},\beta_{2}\right)=\frac{1}{M}\sigma_{g2}^{2}I_{M}\\ cov\left(\beta_{1},\beta_{2}\right)=\frac{1}{M}\gamma_{g}I_{M}. \end{array}$$

Here, $I_{M}$ is an M×M identity matrix, $\sigma_{gt}^{2}$ denotes the genetic variance associated with trait *t* (t∈{1,2}), and $\gamma_{g}$ denotes the genetic covariance. For the environmental effects, we assume $E\left[\varepsilon_{1}\right]=0,E\left[\varepsilon_{2}\right]=0$ and

(Equation 3) $$\begin{array}{l} cov\left(\varepsilon_{1},\varepsilon_{1}\right)=\sigma_{e1}^{2}I_{N}\\ cov\left(\varepsilon_{2},\varepsilon_{2}\right)=\sigma_{e2}^{2}I_{N}\\ cov\left(\varepsilon_{1},\varepsilon_{2}\right)=\gamma_{e}C. \end{array}$$

The genetic correlation parameter $\rho_{g}$ is defined as $\rho_{g}\equiv \gamma_{g}/\sqrt{\sigma_{g1}^{2}}\sqrt{\sigma_{g2}^{2}}$. Importantly, SCORE does not make additional assumptions on the distribution of the genetic effect sizes or the environmental noise.

### Method of moments (MoM)

SCORE uses a method of moments (MoM) estimator to estimate the parameters $\left(\gamma_{g},\gamma_{e},\sigma_{g1}^{2},\sigma_{g2}^{2},\sigma_{e1}^{2},\sigma_{e2}^{2}\right)$.

Because the mean of $y_{1}$ and $y_{2}$ are zero, we focus on the covariance. The population covariance of the concatenated phenotypes $y\equiv {\left[y_{1}^{T},y_{2}^{T}\right]}^{T}$ is now

(Equation 4) $$cov\left(y\right)=E\left[yy^{T}\right]-E\left[y\right]E{\left[y\right]}^{T}=\left[\begin{array}{ll} \sigma_{g1}^{2}K_{1} & \gamma_{g}K_{A}\\ \gamma_{g}K_{A}^{T} & \sigma_{g2}^{2}K_{2} \end{array}\right]+\left[\begin{array}{ll} \sigma_{e1}^{2}I_{N_{1}} & \gamma_{e}C\\ \gamma_{e}C^{T} & \sigma_{e2}^{2}I_{N_{2}} \end{array}\right].$$

Here, $K_{1}=\left(X_{1}X_{1}^{T}/M\right)$ is the GRM for the samples observed for the first trait, while $K_{2}=\left(X_{2}X_{2}^{T}/M\right)$ is the GRM for the samples for the second trait and $K_{A}=\left(X_{1}X_{2}^{T}/M\right)$ is the GRM for pairs of samples across traits.

We obtain the MoM estimator by minimizing the sum of squared differences between the population and empirical covariances:

(Equation 5) $$\left(\hat{\gamma_{g}},\hat{\gamma_{e}},\hat{\sigma_{g1}^{2}},\hat{\sigma_{g2}^{2}},\hat{\sigma_{e1}^{2}},\hat{\sigma_{e2}^{2}}\right)=argmin_{\gamma_{g},\gamma_{e},\sigma_{g1}^{2},\sigma_{g2}^{2},\sigma_{e1}^{2},\sigma_{e2}^{2}}{\left\|yy^{T}-\left(\left[\begin{array}{ll} \sigma_{g1}^{2}K_{1} & \gamma_{g}K_{A}\\ \gamma_{g}K_{A}^{T} & \sigma_{g2}^{2}K_{2} \end{array}\right]+\left[\begin{array}{ll} \sigma_{e1}^{2}I_{N_{1}} & \gamma_{e}C\\ \gamma_{e}C^{T} & \sigma_{e2}^{2}I_{N_{2}} \end{array}\right]\right)\right\|}_{F}^{2}.$$

The MoM estimator for the genetic covariance satisfies the normal equations

(Equation 6) $$\left[\begin{array}{ll} tr\left(K_{A}K_{A}^{\text{T}}\right) & tr\left(K_{C}\right)\\ tr\left(K_{C}\right) & N \end{array}\right]\left[\begin{array}{l} \hat{\gamma_{g}}\\ \hat{\gamma_{e}} \end{array}\right]=\left[\begin{array}{l} y_{1}^{T}K_{A}y_{2}\\ y_{1}^{T}Cy_{2} \end{array}\right],$$

where $K_{C}=\left(X_{1}X_{2}^{T}C^{T}/M\right)$. Given the coefficients of the normal equations, we can solve analytically for $\hat{\gamma_{g}}$ and $\hat{\gamma_{e}}$.

Given MoM estimates of the variance components, the MoM estimate of the genetic correlation is given by the plug-in estimate:

(Equation 7) $$\hat{\rho_{g}}=\frac{\hat{\gamma_{g}}}{\sqrt{\hat{\sigma_{g1}^{2}}}\sqrt{\hat{\sigma_{g2}^{2}}}}.$$

### SCORE: Scalable genetic correlation estimator

Naive computation of the MoM estimate of genetic covariance requires computing $tr\left(K_{A}K_{A}^{\text{T}}\right)$, which requires $O\left(N_{1}N_{2}M\right)$ operations, where $N_{1},N_{2}$ are the sample size of each of the traits.

To overcome this computational bottleneck, we replace $tr\left(K_{A}K_{A}^{\text{T}}\right)$ with an unbiased randomized estimate: $\hat{tr\left(K_{A}K_{A}^{\text{T}}\right)}$.^16^

Given *B* random vectors, $z_{1},\ldots ,z_{B}$, $z_{b}\in R^{N_{2}},b\in 1\ldots B$ drawn independently from a distribution with zero mean and identity covariance, our estimator is given by:

$$L_{B}=\hat{tr\left(K_{A}K_{A}^{\text{T}}\right)}=\frac{1}{B}\frac{1}{M^{2}}\sum_{b}{\left\|X_{1}X_{2}^{T}z_{b}\right\|}_{2}^{2}.$$

We obtain the SCORE estimator $\left(\tilde{\gamma_{g}},\tilde{\gamma_{e}}\right)$ by solving Equation 6 by replacing $tr\left(K_{A}K_{A}^{\text{T}}\right)$ with $L_{B}$.

$$\left[\begin{array}{ll} L_{B} & tr\left(K_{C}\right)\\ tr\left(K_{C}\right) & N \end{array}\right]\left[\begin{array}{l} \tilde{\gamma_{g}}\\ \tilde{\gamma_{e}} \end{array}\right]=\left[\begin{array}{l} y_{1}^{T}K_{A}y_{2}\\ y_{1}^{T}Cy_{2} \end{array}\right].$$

Here, $tr\left(K_{C}\right)$ denotes the sum of the squared genotypes for individuals measured on both traits so that $tr\left(K_{C}\right)$ can be computed in time: O(MN). Computing $L_{B}$ requires multiplying the genotype matrices $X_{1}$ and $X_{2}$ with *B* vectors resulting in a runtime of $O\left(max\left(N_{1},N_{2}\right)MB\right)$. Leveraging the fact that each element of the genotype matrix takes values in the set {0,1,2}, $L_{B}$ can be computed in time $O\left(max\left(\left(N_{1}/max\left(log_{3}N_{1},log_{3}M\right)\right),\left(N_{2}/max\left(log_{3}N_{2},log_{3}M\right)\right)\right)MB\right)$ ^17^ (while the standardized genotypes are real-valued, SCORE computes the equivalent quantities by operating on the unstandardized genotype matrix to be able to leverage its discrete entries followed by subtracting the product of the mean of a SNP and random vectors and scaling by minor allele frequency [MAF]). Combined with our previous efficient estimators of the genetic variance components,^18^^,^^19^ we obtain an efficient estimator of $\rho_{g}$.

In the setting where the two traits are measured on the same set of individuals, we can estimate the $\rho_{g}$ directly without the need for separately estimating $\gamma_{g}$, $\sigma_{g1}^{2}$, and $\sigma_{g2}^{2}$. This estimator does not rely on any randomized approximations and can be computed in time $O\left(NM/max\left({\text{log}}_{3}N,{\text{log}}_{3}M\right)\right)$. We term this modification SCORE−OVERLAP (supplemental material and methods).

### Simulations to assess accuracy

We performed simulations on a subset of 5,000 unrelated white British individuals from the UK Biobank so that all methods compared could be run in a reasonable time. Our simulations used 305,630 SNPs with MAF above 1% (we chose these SNPs because these were also used for benchmarking the HDL^12^ method and had reference eigenvectors available).

Given the genotypes, we simulated pairs of traits under varying genetic architectures. Our first set of architectures assume an infinitesimal model (where all variants have a non-zero effect on both traits). We varied genetic correlation $\rho_{g}$ across {0,0.2,0.5,0.8} and the heritability of the pair of traits, $\left(h_{1}^{2},h_{2}^{2}\right)$, across values of {(0.1,0.2),(0.2,0.6),(0.5,0.5),(0.6,0.8)} corresponding to the situation where both traits have low heritability, one trait has low while the other has moderate heritability, both traits have moderate heritability, and both have high heritability.

Our next set of non-infinitesimal architectures explore traits with medium polygenicity and low polygenicity. For each SNP *m*, we specify a causal status, $c_{m}$, which is a 2×1 vector with entries taking values in {0,1} according to whether SNP *m* has a non-zero effect on each of the two traits. For medium polygenicity, causal status at SNP *m* is drawn independently according to the following distribution: $P\left(c_{m}=\left[\begin{array}{l} 1\\ 1 \end{array}\right]\right)=0.1$, $P\left(c_{m}=\left[\begin{array}{l} 0\\ 1 \end{array}\right]\right)=P\left(c_{m}=\left[\begin{array}{l} 1\\ 0 \end{array}\right]\right)=0.2$, and $P\left(c_{m}=\left[\begin{array}{l} 0\\ 0 \end{array}\right]\right)=0.5$.

The effect size $\beta_{m}$ of SNP *m* on each trait is drawn from the following distribution:

$$\beta_{m}|c_{m}=\left[\begin{array}{l} 1\\ 1 \end{array}\right]∼N(0,\left[\begin{array}{ll} \frac{\sigma_{g1}^{2}}{0.3M} & \gamma_{g}\\ \gamma_{g} & \frac{\sigma_{g2}^{2}}{0.3M} \end{array}\right],\beta_{m}|c_{m}=\left[\begin{array}{l} 1\\ 0 \end{array}\right]∼N(0,\left[\begin{array}{ll} \frac{\sigma_{g1}^{2}}{0.3M} & 0\\ 0 & 0 \end{array}\right],\beta_{m}|c_{m}=\left[\begin{array}{l} 0\\ 1 \end{array}\right]∼N(0,\left[\begin{array}{ll} 0 & 0\\ 0 & \frac{\sigma_{g2}^{2}}{0.3M} \end{array}\right]$$

For low polygenicity, we set the probability $P\left(c_{m}=\left[\begin{array}{l} 1\\ 1 \end{array}\right]\right)=0.01$, $P\left(c_{m}=\left[\begin{array}{l} 0\\ 1 \end{array}\right]\right)=P\left(c_{m}=\left[\begin{array}{l} 1\\ 0 \end{array}\right]\right)=0.05$, and $P\left(c_{m}=\left[\begin{array}{l} 0\\ 0 \end{array}\right]\right)=0.89$.

The effect size $\beta_{m}$ for genetic variant *m* on both traits are drawn from the following distribution:

$$\beta_{m}|c_{m}=\left[\begin{array}{l} 1\\ 1 \end{array}\right]∼N(0,\left[\begin{array}{ll} \frac{\sigma_{g1}^{2}}{0.06M} & \gamma_{g}\\ \gamma_{g} & \frac{\sigma_{g2}^{2}}{0.06M} \end{array}\right],\beta_{m}|c_{m}=\left[\begin{array}{l} 1\\ 0 \end{array}\right]∼N(0,\left[\begin{array}{ll} \frac{\sigma_{g1}^{2}}{0.06M} & 0\\ 0 & 0 \end{array}\right],\beta_{m}|c_{m}=\left[\begin{array}{l} 0\\ 1 \end{array}\right]∼N(0,\left[\begin{array}{ll} 0 & 0\\ 0 & \frac{\sigma_{g2}^{2}}{0.06M} \end{array}\right].$$

We vary $\gamma_{g}$ across {0,0.2,0.5,0.8}. Under this model, the true total expected genome-wide genetic correlation for medium polygenicity is {0,0.06,0.15,0.24} and {0,0.0024,0.03,0.048} for low polygenicity. Unless specified otherwise, we assume complete sample overlap and no environmental correlation, set the environmental variance so that the trait variance is 1, and simulate a total of 100 replicates for each architecture.

### Simulations to assess the impact of sample overlap

We simulated traits under an infinitesimal architecture with $\left(h_{1}^{2},h_{2}^{2}\right)=\left(0.2,0.6\right)$ and $\rho_{g}=$ 0.5. For each trait, we fixed the sample size to 5,000 and varied the proportion of sample overlap across {0,0.2,0.5,0.8,1} (ranging from no overlap to complete overlap). Specifically, for overlap proportion equal to 0, we have 5,000 samples with observations on the first trait and a distinct set of 5,000 samples with observations on the second trait. For overlap proportion equal to 1, we have 5,000 samples with observations on both traits. We estimated genetic correlation with SCORE, LDSC, and GCTA-GREML.

### Simulations to assess accuracy for binary traits

Given 291,273 unrelated white British individuals in the UK Biobank measured on 459,792 genetic variants, we simulated pairs of traits under an infinitesimal architecture setting $\left(h_{1}^{2},h_{2}^{2}\right)=\left(0.272,0.12\right)$ and $\rho_{g}=-0.23$ while varying the environmental correlation across {0.04,−0.04,0}.

To simulate binary traits, we converted the second trait to a binary trait by thresholding the underlying continuous trait such that the prevalence varied across {0.01%,0.5%,1%}.

### Data processing

LD scores were computed from 305,630 SNPs chosen for the simulations. The LD scores were computed from a random subset of 50,000 individuals in the UK Biobank (the individuals used in our simulations were a subset of the 50,000 individuals used for computation of LD score). For analysis of UK Biobank data, LD scores were computed on 459,792 common SNPs (MAF >1%) present on the UK Biobank Axiom array. LD scores were computed with flags −−l2 and −−ld−wind−kb2000.0.

Summary statistics input to LDSC were generated with PLINK. We used linear regression to generate summary statistics for continuous traits and categorical traits and logistic regression for binary traits. In computing summary statistics for traits in the UK Biobank, we include the following covariates: age, gender, principal components 1–10, assessment center, and genotype measurement batch. We used the same covariates as input to SCORE.

We ran LDSC under default settings with an unconstrained intercept. In addition to summary statistics, HDL requires eigenvectors of the LD matrix. We used the eigenvectors that preserve 90% of the variance of the LD blocks that were released by the study authors. Computation of the eigenvectors used the same set of genetic variants as our simulations and 336,000 samples in the UK Biobank.^12^

### Quality control of UK Biobank data

We restricted our analysis to SNPs genotyped on the UK Biobank Axiom array, filtering out markers that had high missingness rate (>1%) and low MAF (<1%), and we exclude the major histocompatibility complex (MHC) region. Moreover, SNPs that fail the Hardy-Weinberg equilibrium (HWE) test at significance threshold $10^{-7}$ were removed. We also filter the samples that have a genetic kinship with any other sample (samples having any relatives in the dataset using the field 22021: “Genetic kinship to other participants”) and restricted the study to samples with self-reported British white ancestry (field 21000 with coding 1001). After quality control, we obtained 291,273 individuals and 454,207 SNPs.

We performed similar quality control on the imputed genotypes in the UK Biobank: filtering out markers with high missingness rate (>1%), low MAF (<1%), and HWE p value $<1\times 10^{-7}$ and that fall within the MHC region. After quality control, we obtained 4,824,392 SNPs.

We chose traits that have missingness <30% and disease traits with prevalence larger than 0.5%, resulting in a total of 40 phenotypes consisting of 14 binary traits, three categorical traits, and 23 continuous traits. The 40 phenotypes could be classified into nine groups: glucose metabolism and diabetes, socioeconomic and general medical information, environmental factor, coronary artery disease related, autoimmune disorders, psychiatric disorders, anthropometric, blood pressure and circulatory, and lipid metabolism (Table S10).

## Results

### Accuracy and robustness of SCORE in simulations

We performed simulations to compare the accuracy of SCORE to other estimators of genetic correlation under different genetic architectures. Specifically, we compared SCORE to methods that use individual data (bi-variate GREML,^2^ bi-variate Haseman-Elston regression) and methods that rely on summary statistics (LD-score regression [LDSC]^7^ and HDL^12^). Bi-variate GREML (GCTA-GREML) and Haseman-Elston regression (GCTA-HE) are implemented in the GCTA software. LDSC is a widely used method to estimate genetic correlation when only summary statistics from GWASs on pairs of traits are available. HDL is a recent summary-statistics-based method that has been shown to obtain improved statistical efficiency relative to LDSC given additional information about LD. We ran all methods on the same set of SNPs to ensure a fair comparison.

We performed simulations to assess the estimation accuracy of each method by using a subset of 5,000 unrelated white British individuals in the UK Biobank so that all the methods could be run in a reasonable time. Unless otherwise specified, all our simulations used 305,630 SNPs with MAF above 1%. We simulated pairs of traits under a total of 48 genetic architectures: varying heritability of the pair of traits $\left(h_{1}^{2},h_{2}^{2}\right)$, genetic correlation $\left(\rho_{g}\right)$, and polygenicity (proportion of causal variants shared and unique to each trait).

The simulations assume that the two traits are measured on the same set of individuals so that both SCORE and SCORE-OVERLAP can be applied in this setting. Because SCORE is a randomized estimator, we first examined the choice of the number of random vectors (*B*) on the estimates of $\rho_{g}$. First, we confirmed that SCORE (with B=10 and B=100 random vectors) and SCORE-OVERLAP yield nearly identical results across the 48 architectures (Table S1). Second, we ran SCORE with different choices of B=10 random vectors on a single replicate that was simulated under the infinitesimal architecture with trait heritability $\left(h_{1}^{2},h_{2}^{2}\right)=\left(0.2,0.6\right)$, and $\rho_{g}=0.5$. We observe that the standard deviation of $\rho_{g}$ estimates across choices of random vectors is about 18% of the total standard error (SE), indicating that the choice of B=10 makes a modest contribution to variability in $\rho_{g}$ estimates. These results lead us to use SCORE with B=10 as our default.

**Figure 1:**
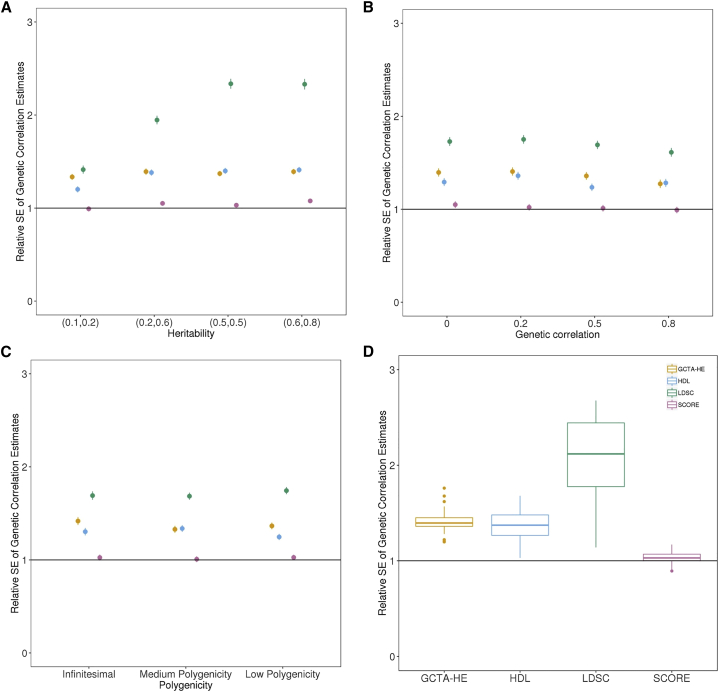
Comparison of the estimates of genetic correlation from SCORE with GCTA-GREML, GCTA-HE, LDSC, and HDL (N=5,000 unrelated individuals, M=305,630 SNPs) (A–D) We simulated pairs of traits under 48 genetic architectures (with varying heritability, genetic correlation, and polygenicity). We plot the standard error (SE) of each method relative to GCTA-GREML. (A), (B), and (C) display the standard error (SE) of each method relative to GCTA-GREML as a function of heritability, genetic correlation, and polygenicity, while (D) summarizes the relative SE across all architectures (see the simulations to assess accuracy section of material and methods). We ran LDSC with in-sample LD and HDL with eigenvectors that preserve 90% variance (see the data processing section of material and methods). We estimate the standard error of the relative SE by using Jackknife (error bars denote 1 standard error).

Across the 48 architectures that we examined, the SE of SCORE ranges from 0.89 to 1.17 relative to the SE of GCTA-GREML; the SE of SCORE is 2.5% higher than that of GCTA-GREML on average (Figures 1). Interestingly, GCTA-HE tends to have an SE that is 1.38 times that of SCORE on average (range 1.2 to 1.6). Compared to methods that rely on summary statistics, LDSC has 1.8 times the SE of SCORE on average (range 1.08 to 2.63), while the SE of HDL relative to SCORE is 1.24 (range 1.05 to 1.65) (Figure 1, Table S2). The reduction in the SE of SCORE relative to the summary-statistic-based methods is equivalent to a 3.24-fold increase in sample size over LDSC and a 1.56-fold increase in sample size over HDL on average. We find that the accuracy of SCORE relative to the other methods is consistent across infinitesimal (Figure S1) and non-infinitesimal architectures (Figure S2 for medium and Figure S3 for low polygenicity; the bias, SE, and mean squared error (MSE) of each of the methods is listed in Tables S3, S4, and S5). We additionally investigated the accuracy of each of the methods across a larger sample size of 10,000 unrelated white British individuals chosen so that it was computationally feasible to run all methods, including GCTA-GREML and GCTA-HE. Under a non-infinitesimal architecture with medium polygenicity, $\rho_{g}=0.5$ and $\left(h_{1}^{2},h_{2}^{2}\right)=\left(0.2,0.6\right)$. In this larger sample size, we observe that SEs of GCTA-GREML, GCTA-HE, and LDSC are 0.97,1.54, and 2.85 times that of SCORE, respectively, consistent with our results on a N=5,000.

We performed additional simulations to investigate the robustness of SCORE. First, we investigated the impact of sample overlap under an infinitesimal genetic architecture with $\rho_{g}=0.5$. The SE of SCORE relative to GCTA-GREML and LDSC remains stable as a function of sample overlap (Figure S4 and Table S6 for the bias, SE, and MSE of SCORE, GCTA-GREML, and LDSC as a function of sample overlap). Second, we verified that the Jackknife standard error estimate used in SCORE is generally accurate while being conservative for low trait heritability (Table S7). Third, we verified the false positive rate of SCORE is controlled in simulations where $\rho_{g}$ is zero. For each of 100 replicates in a given genetic architecture, we computed p values for the two-tailed test of the null hypothesis that $\rho_{g}$ is zero. Averaging across all architectures, we observe that the false positive rate (the fraction of simulations for which the p value <0.05) is 0.04 (Table S8). We additionally verified that the false positive rate in a large-scale simulation (N=291,273) with different prevalences if one of the traits is binary and the situation where both traits are continuous. We observe that the false positive rate is not affected by the prevalence of binary trait (Table S8). Finally, we evaluated the accuracy of SCORE when applied to pairs of traits where one of the traits is binary while the other is continuous. We observe that the $\rho_{g}$ estimates of SCORE are unbiased across the range of prevalence of the binary trait (Table S9). Further, the estimates of $\rho_{g}$ obtained by SCORE tend to have relatively low SE provided the prevalence of the trait is greater than 0.5% (Table S9) so that we recommend applying SCORE to traits whose prevalence is no less than 0.5%.

### Computational efficiency

**Figure 2:**
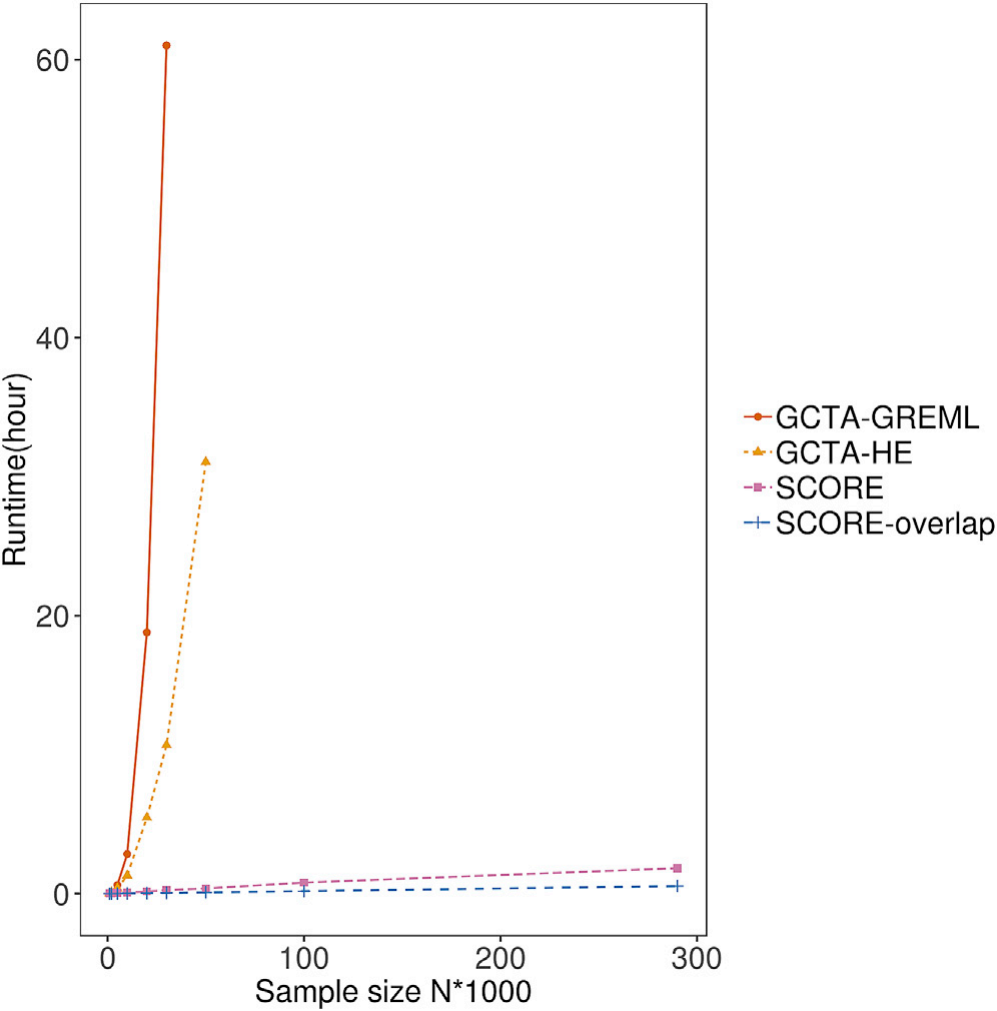
Comparison of the runtime of SCORE with GCTA-GREML and GCTA-HE as a function of the number of samples The samples were obtained as subsets of unrelated, white British individuals in the UK Biobank. We plot the runtime of both SCORE (that can handle any degree of sample overlap) and its variant, SCORE-OVERLAP (designed for 100% sample overlap). SCORE runs in a few h on the largest dataset of 291,273 individuals and 454,207 SNPs.

We investigated the computational efficiency of SCORE relative to GCTA-GREML and GCTA-HE. The runtime and memory usage of summary statistic methods (LDSC and HDL) depend on the time needed to compute LD scores and summary statistics of each trait. In addition, HDL also requires the computation of the singular value decomposition (SVD) of LD matrices, which is a computationally expensive step. Thus, we do not include runtimes for LDSC and HDL in these comparisons. We varied the number of individuals, while the number of SNPs was fixed at 454,207. Figure 2 shows that GCTA-GREML and GCTA-HE could not scale beyond sample sizes greater than 100,000 because of the requirement of computing and operating on a GRM (we extrapolate the runtime of GCTA-GREML and GCTA-HE to be about 340 days and 44 days on the set of 291,273 unrelated white British individuals in the UK Biobank). On the other hand, SCORE ran in about 1.5 h on the set of 291,273 individuals by using partial overlap mode with B=10 random vectors, while the SCORE-OVERLAP variant ran in about 1 h on the same dataset.

### Application of SCORE to UK Biobank

**Figure 3:**
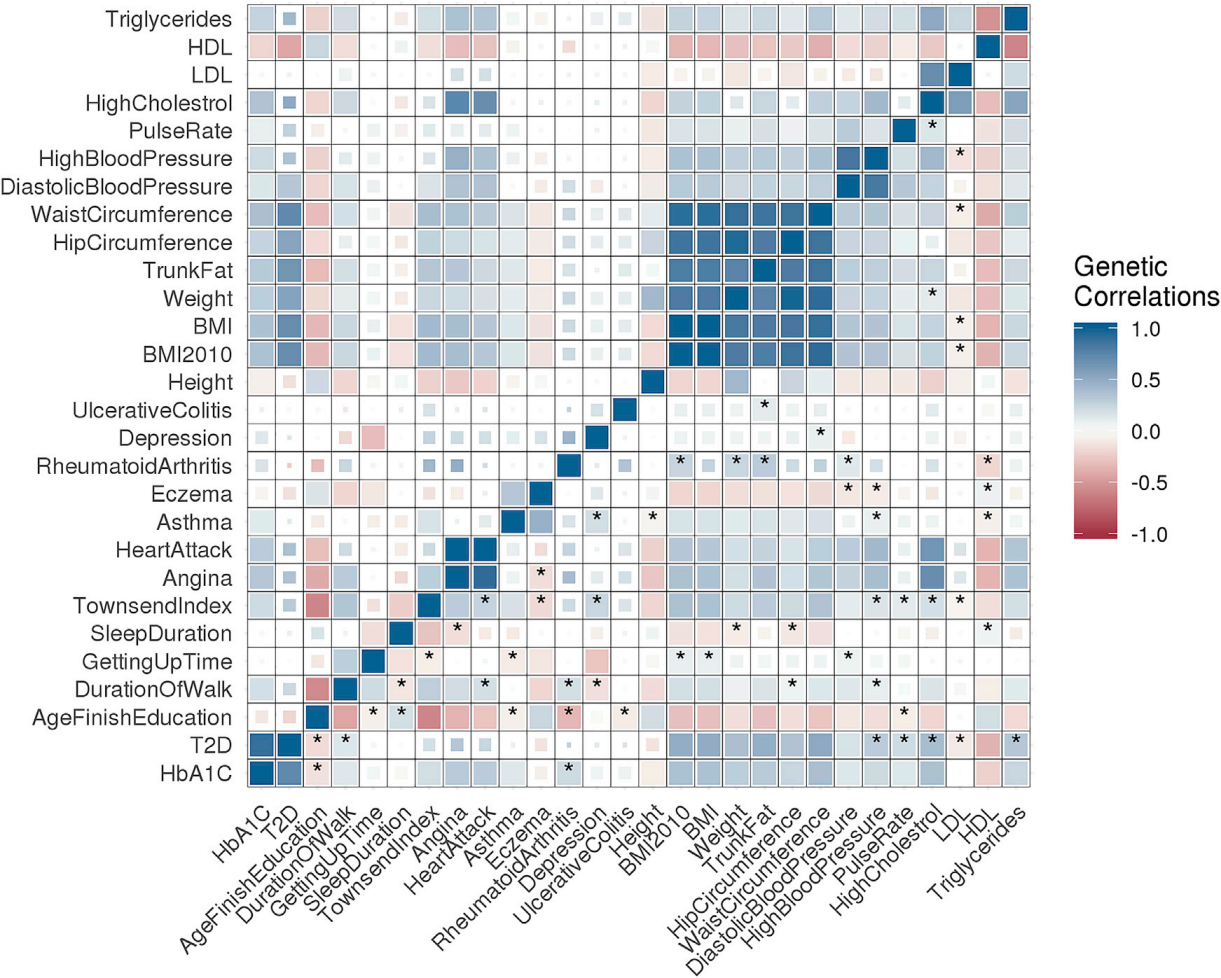
Genetic correlation estimates in the UK Biobank We plot the genetic correlation estimates from SCORE (bottom triangle) and LDSC (upper triangle) across pairs of 28 phenotypes. Larger filled squares correspond to significant pairs after Bonferroni correction at a 5% significance level, while smaller squares correspond to pairs that are significant at a 5% significance level but are not significant after accounting for multiple testing. Star indicates pairs that are found to be significant by SCORE but not by LDSC.

We applied SCORE to estimate $\rho_{g}$ for pairs of phenotypes in the UK Biobank across 291,273 unrelated white British individuals and 454,207 SNPs (material and methods). We compared the $\rho_{g}$ estimates obtained by LDSC versus SCORE for a subset of 28 traits in which LDSC produced valid estimates, i.e., traits for which none of the $\rho_{g}$ estimates were N/A (Figure 3). While the point estimates of $\rho_{g}$ from the two methods are highly concordant (Pearson correlation r=0.95), the SE of LDSC is about 1.57 times that of SCORE on average, which is equivalent to a 2.46-fold increase in sample size via SCORE (see Figures S5 and S6). In total, 192 pairs of traits were detected to have a significant non-zero $\rho_{g}$ by both SCORE and LDSC after Bonferroni correction for all pairs across the original set of 40 phenotypes (p<0.05/780). Consistent with its reduced SE, SCORE found 58 pairs with significant $\rho_{g}$ after Bonferroni correction that were not detected as significant by LDSC (p<(0.05/780); stars in Figure 3). We conclude that SCORE obtains improved power to identify statistically significant genetic correlations relative to LDSC.

We obtain concordant results when analyzing all pairs in our initial set of 40 traits. Although the point estimates of SCORE and LDSC are highly correlated (Pearson correlation r=0.96), the SE of LDSC is about 1.8 times that of SCORE on average, equivalent to a 3.24-fold increase in the sample size. In this setting, SCORE found 200 additional pairs of traits over LDSC (beyond the 245 pairs identified by both), while LDSC detected one pair as significant that SCORE did not detect as significant (Figure S7). To understand the impact of random vectors, we repeated our analysis with a different set of random vectors and observed that the Pearson correlation of $\rho_{g}$ estimates with the two sets is 0.999 (Figure S11).

We also analyzed all pairs in our initial set of 40 traits with HDL by using the set of 305,630 SNPs for which reference eigenvectors are available.^12^ The SE of HDL is about 2.53 times that of SCORE on average, which is equivalent to a 6.4-fold increase in the sample size (HDL failed to converge for 11% of the pairs where at least one of the traits is binary). Among these pairs, SCORE found 171 additional pairs of traits over HDL (beyond the 239 pairs identified by both), while HDL detected 14 pairs as significant that SCORE did not detect as significant. The summary of SE ratio of HDL and SCORE is shown in Figure S8.

To gain further insights into SCORE, we examined the SE of $\rho_{g}$ estimates for pairs of traits according to whether the traits were both binary, both quantitative, or had one member of the pair that was binary while the other was quantitative. The SE is largest when both traits are binary, intermediate when one of the traits is binary, and lowest when both traits are quantitative (average SE: 0.082, 0.035, and 0.02, respectively; Figure S9). We note that the SE increases when the prevalence of the binary trait decreases: the mean SE is 0.017 when the binary trait has prevalence >25%, while the mean SE is 0.047 for pairs in which the binary trait has prevalence <5% (Figure S10).

We also applied SCORE to imputed genotypes in 291,273 unrelated white British individuals and 4,824,392 SNPs (MAF >1%). SCORE required about 19 h to analyze a single pair of traits for imputed SNPs while requiring about 1.5 h on array SNPs (scaling linearly with the number of variants). Because SCORE uses a streaming approach that does not require all SNPs to be stored in memory, it is memory efficient, requiring about 2.3 GB to analyze imputed data. The estimates of $\rho_{g}$ are largely concordant across array and imputed SNPs (Pearson correlation of the $\rho_{g}$ point estimates with two sets of SNPs is 0.973). We found 423 trait pairs that have significant non-zero $\rho_{g}$ estimates (after Bonferroni correction) across both imputed and array genotypes, while 19 pairs are significant only in the analysis of imputed genotypes and 22 pairs are significant in the analysis of array genotypes (Figure S12).

To further illustrate its utility, we applied SCORE to estimate genetic correlation between coronary-artery-disease-related traits included in our set of 40 traits (angina and heart attack) and serum biomarkers (alanine [ALT] and aspartate aminotransferase [AST]). Serum liver enzyme levels, including ALT and AST, are markers of liver health and hepatic dysfunction, and they have been shown to be associated with cardiovascular disease,^20^, ^21^, ^22^ although the strength and consistency has varied among the studies.^20^ We observed significant positive $\rho_{g}$ between ALT/AST and the two coronary-artery-disease-related traits (0.257±0.04 and 0.169±0.032 for angina with ALT and AST, respectively; 0.239±0.053 and 0.148±0.04 for heart attack with ALT and AST, respectively). Our finding of significant positive $\rho_{g}$ suggests that hepatic dysfunction (higher serum levels of ALT and AST) and coronary artery disease have a shared genetic component.

## Discussion

We have described SCORE, a scalable and accurate estimator of genetic correlation. We observe that the estimates of genetic correlation obtained by SCORE have accuracy comparable to GCTA-GREML^13^ while being scalable to biobank-scale data. SCORE can estimate the genetic correlation across pairs of traits when applied to ≈500K common SNPs measured on ≈300K unrelated white British individuals in the UK Biobank within a few h. In simulations, we showed that, compared to summary-statistic methods, SCORE obtains a reduction in the average standard error of 44% relative to LDSC and 20% relative to HDL, equivalent to a 3.24-fold and 1.56-fold increase in sample size. In application to 780 pairs of traits in the UK Biobank, SCORE discovered 200 pairs of traits with significant genetic correlation (after correcting for multiple testing) that were not discovered by LDSC. In application to 780 pairs, SCORE discovered 171 pairs of traits with significant genetic correlation (after correcting for multiple testing) that were not discovered by HDL, while HDL discovered 14 significant pairs not discovered by SCORE. It is plausible that the results of HDL might be altered by the computation of eigenvectors from the analyzed genotypes, although such an analysis can be computationally expensive

The statistical accuracy gain of SCORE relative to LDSC and HDL can be attributed to several factors. LDSC does not use all the available covariances among the summary statistics choosing to only model the variance. The LD information as summarized by the LD scores involve a number of approximations. Typically, LD scores are computed from an external reference panel. Even when in-sample LD is used (as we have here), computational considerations lead to the LD scores’ being computed from a subset of the samples and restricted to SNPs that fall within a fixed-length genomic window. While HDL models the covariance structure among the summary statistics, thereby utilizing additional information relative to LDSC, HDL relies on approximate computations of LD scores like LDSC. To enable computational efficiency, HDL also uses a truncated SVD of the LD score matrix that can potentially further reduce accuracy.

We discuss several limitations of SCORE. First, SCORE requires access to individual genotype and trait data. Summary-statistic methods such as LDSC and HDL have the advantage of being applicable in settings where access to individual-level data can be challenging. While summary-statistic methods also have the advantage of being relatively efficient, it is important to keep in mind that the summary statistics are dependent on specific choices of marker sets and covariates. Applying these methods to different sets of covariates and marker sets requires regenerating the summary statistics (and auxiliary information such as LD score matrices). Second, the model underlying SCORE assumes a quantitative trait. We have shown empirically that SCORE provides accurate estimates of genetic correlation when applied to binary traits provided the traits are not too rare (prevalence >0.5%). It would be of interest to extend SCORE to the setting of binary traits along the lines of the PCGC method.^11^ Finally, while SCORE estimates genome-wide genetic correlation, efficient methods that can partition genetic correlation across genomic annotations can provide additional insights into the shared genetic basis of traits.

## Web resources

GCTA-GREML and GCTA-HE, https://yanglab.westlake.edu.cn/software/gcta/
HDL reference panel, https://github.com/zhenin/HDL/wiki/Reference-panels
HDL software, https://github.com/zhenin/HDL
LDSC software, https://github.com/bulik/ldsc/
PLINK1.9, https://www.cog-genomics.org/plink/2.0
SCORE software, https://github.com/sriramlab/SCORE
UK Biobank, https://www.ukbiobank.ac.uk/

## Supplemental information

Document S1. Figures S1–S12, Tables S1–S10, and supplemental material and methods

Document S2. Article plus supplemental information
