# Supplementary material for: Fast estimation of genetic correlation for biobank-scale data
Source: Am J Hum Genet. 2021 Dec 2;109(1):24–32. doi: 10.1016/j.ajhg.2021.11.015 (PMC8764132; doi:10.1016/j.ajhg.2021.11.015)
Supplement: Document S1. Figures S1–S12, Tables S1–S10, and supplemental material and methods [file mmc1.pdf]

The American Journal of Human Genetics, Volume *109*

Supplemental information

Fast estimation of genetic correlation for biobank-scale data

Yue Wu, Kathryn S. Burch, Andrea Ganna, Päivi Pajukanta, Bogdan Pasaniuc, and Sriram Sankararaman

Supplemental Figures and legends

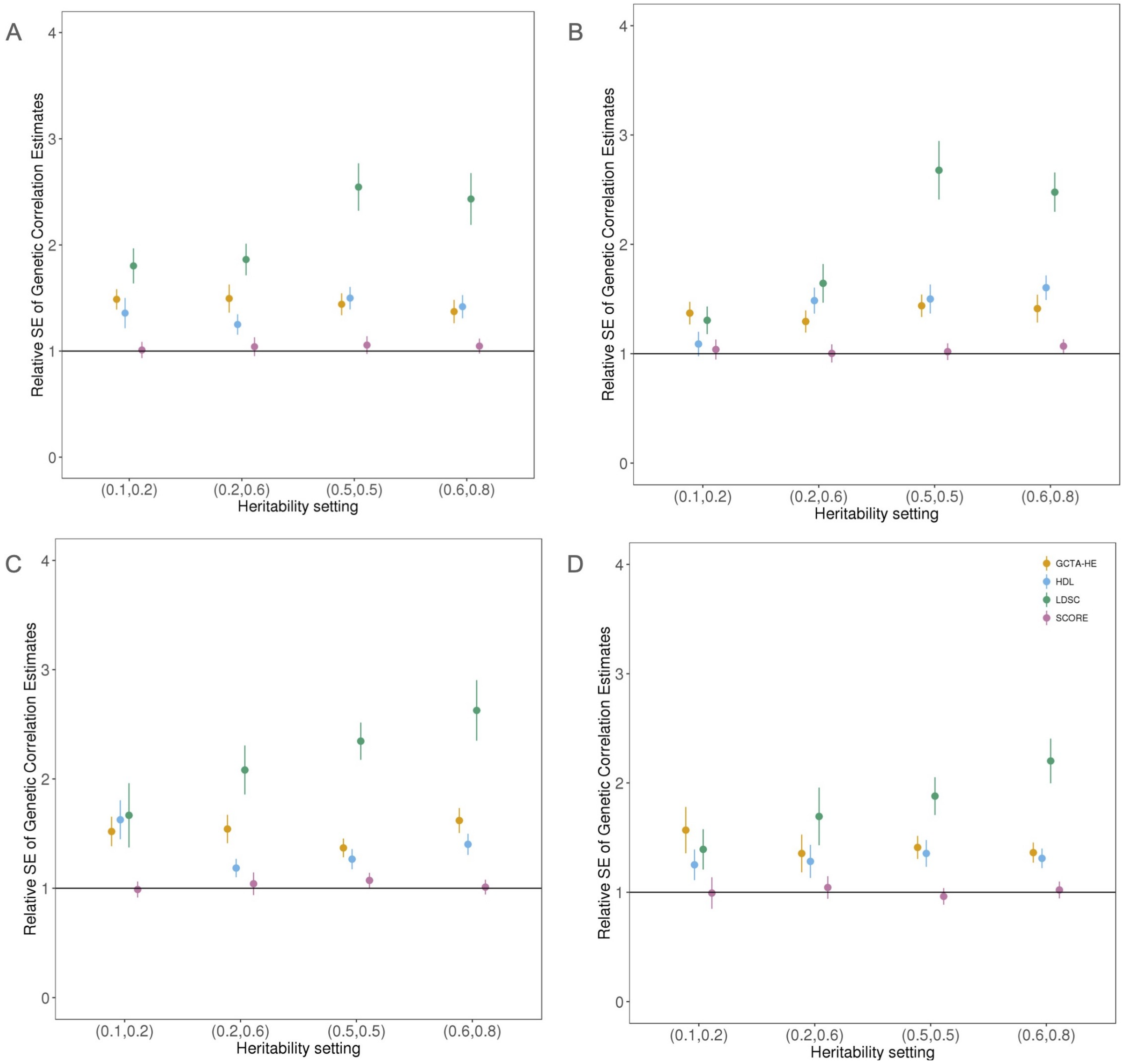

Figure S1: **Comparison of the estimates of genetic correlation from SCORE with GCTA-GREML, GCTA-HE, LDSC, and HDL in small-scale simulations ($N = 5,000$ unrelated individuals, $M = 305,630$ SNPs) under infinitesimal genetic architectures**. We simulated pairs of phenotypes under 16 different infinitesimal genetic architectures. Panel A, B, C, D correspond to a different value of the genetic correlation chosen from the set: $\{0, 0.2, 0.5, 0.8\}$. Within each panel, we varied the SNP heritability for the pair of traits across $\{(0.1, 0.2), (0.2, 0.6), (0.5, 0.5), (0.6, 0.8)\}$ (see Simulations to assess accuracy section of Materials and Methods). We plot the standard error (SE) of each method relative to GCTA-GREML. We estimate the standard error of the relative SE using Jackknife (error bars denote 1 standard error).

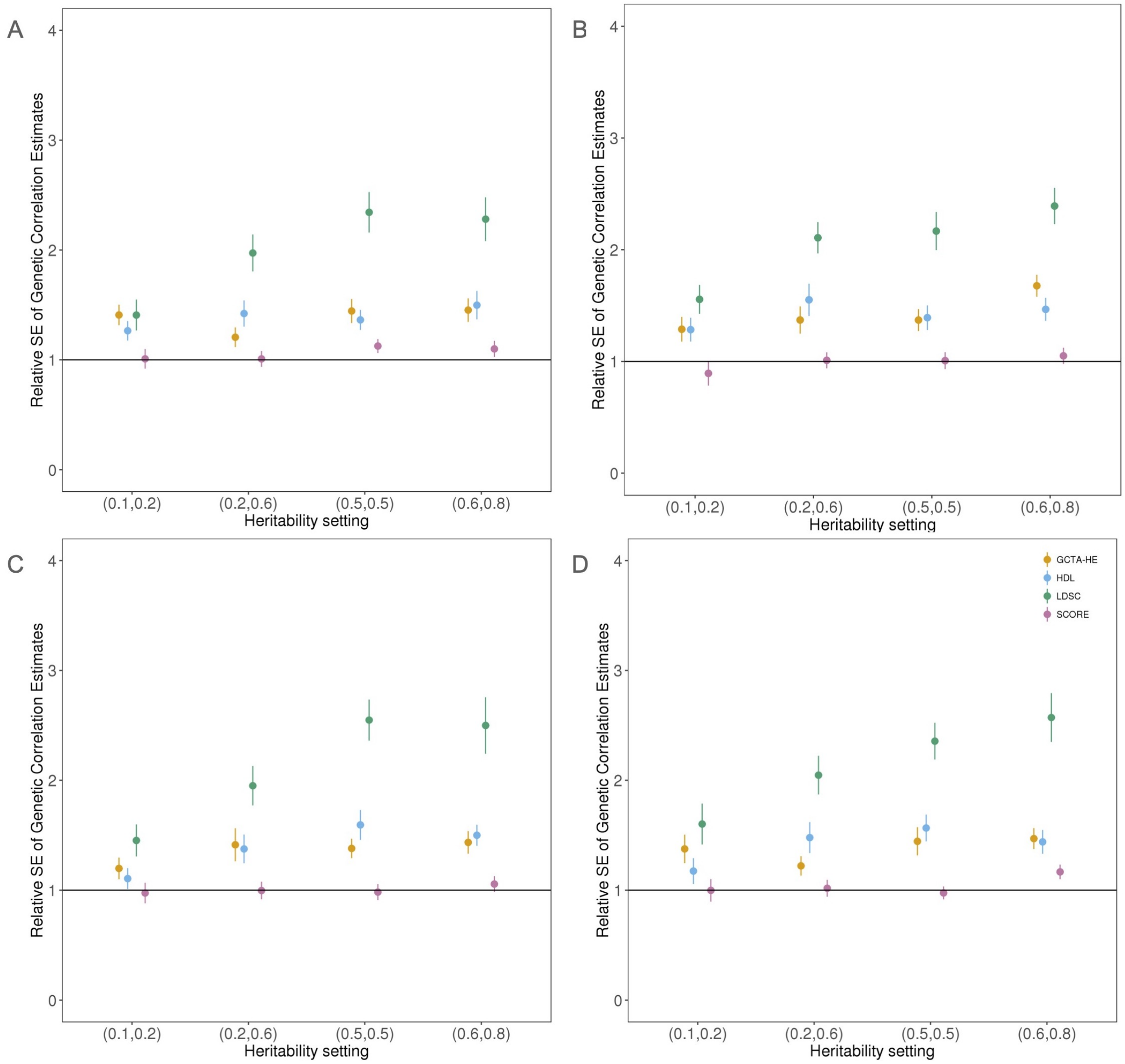

Figure S2: **Comparison of the estimates of genetic correlation from SCORE with GCTA-GREML, GCTA-HE, LDSC, and HDL in small-scale simulations ($N = 5,000$ unrelated individuals, $M = 305,630$ SNPs) under non-infinitesimal architectures with medium polygenicity**. We simulated pairs of phenotypes under 16 different non-infinitesimal genetic architectures. The probability of a variant being causal for both traits is 0.20, and the probability of a variant being causal for exactly one of the trait is 0.10. Panels (A, B, C, D) correspond to a different value of the genetic correlation at SNPs causal for both traits: $\{0, 0.2, 0.5, 0.8\}$. The causal variants are distributed uniformly across the genome. Within each panel, we varied the per-SNP heritability of variants causal for both traits to be proportional to $\{(0.1, 0.2), (0.2, 0.6), (0.5, 0.5), (0.6, 0.8)\}$ (see Simulations to assess accuracy section of Materials and Methods). We plot the SE of each method relative to GCTA-GREML. We ran LDSC with in-sample LD and HDL with eigenvectors that preserve 90% variance. We estimate the standard error of the relative SE using Jackknife (error bars denote 1 standard error).

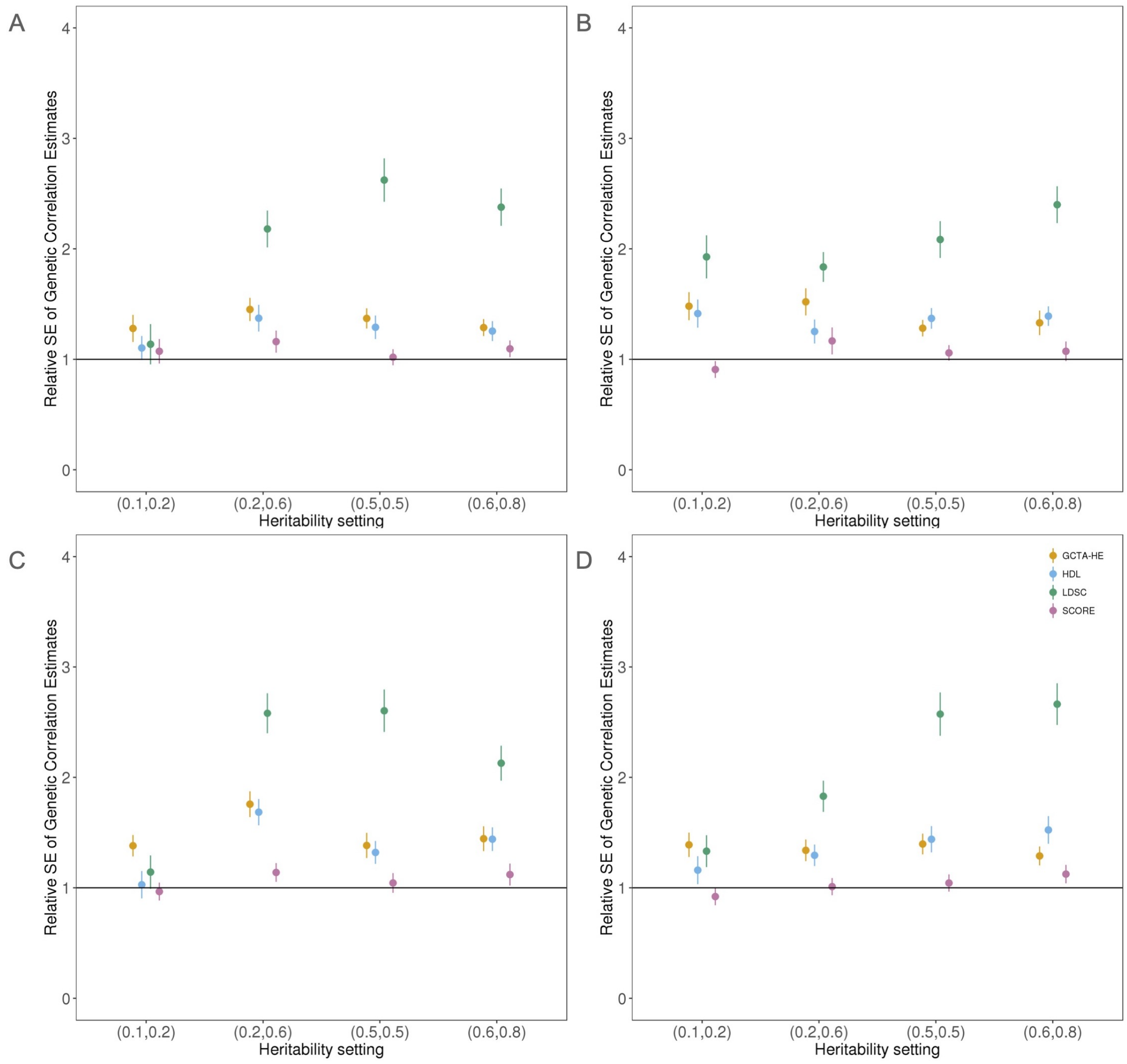

Figure S3: **Comparison of the estimates of genetic correlation from SCORE with GCTA-GREML, GCTA-HE, LDSC, and HDL in small-scale simulations ($N = 5,000$ unrelated individuals, $M = 305,630$ SNPs) under non-infinitesimal architectures with low polygenicity**. We simulated pairs of phenotypes under 16 different non-infinitesimal genetic architectures. The probability of a variant being causal for both traits is 0.01, and the probability of a variant being causal for exactly one of the trait is 0.05. Panels (A, B, C, D) correspond to a different value of the genetic correlation at SNPs causal for both traits: $\{0, 0.2, 0.5, 0.8\}$. The causal variants are distributed uniformly across the genome. Within each panel, we varied the per-SNP heritability of variants causal for both traits to be proportional to $\{(0.1, 0.2), (0.2, 0.6), (0.5, 0.5), (0.6, 0.8)\}$ (see Simulations to assess accuracy section of Materials and Methods). We plot the SE of each method relative to GCTA-GREML. We ran LDSC with in-sample LD and HDL with eigenvectors that preserve 90% variance. We estimate the standard error of the relative SE using Jackknife (error bars denote 1 standard error).

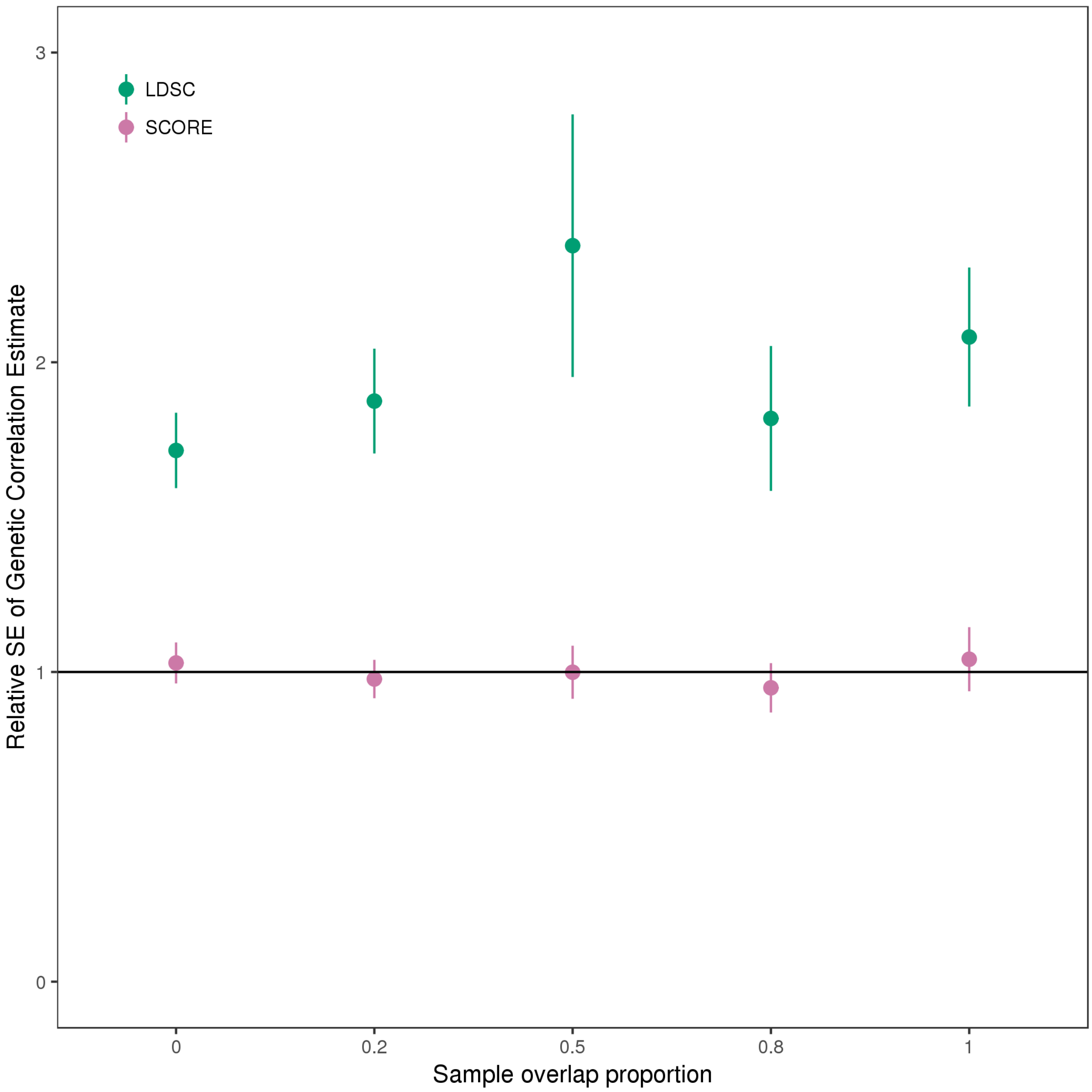

Figure S4: **Comparison of the estimates of genetic correlation from SCORE with GCTA-GREML and LDSC as a function of sample overlap ($M = 305,630$ SNPs).** We vary the proportion of sample overlap across $\{0, 0.2, 0.5, 0.8, 0.1\}$. For sample overlap proportion of 0, we have a total of $10,000$ samples where each sample only has observation on one of the traits. For overlap proportion of 1, we have a total $5,000$ samples with each sample having observations on both traits (see Simulations to assess the impact of sample overlap in Materials and Methods). We report the SE of SCORE and LDSC relative to GCTA-GREML. We ran LDSC with in-sample LD. We estimate the standard error of the relative SE using jackknife.

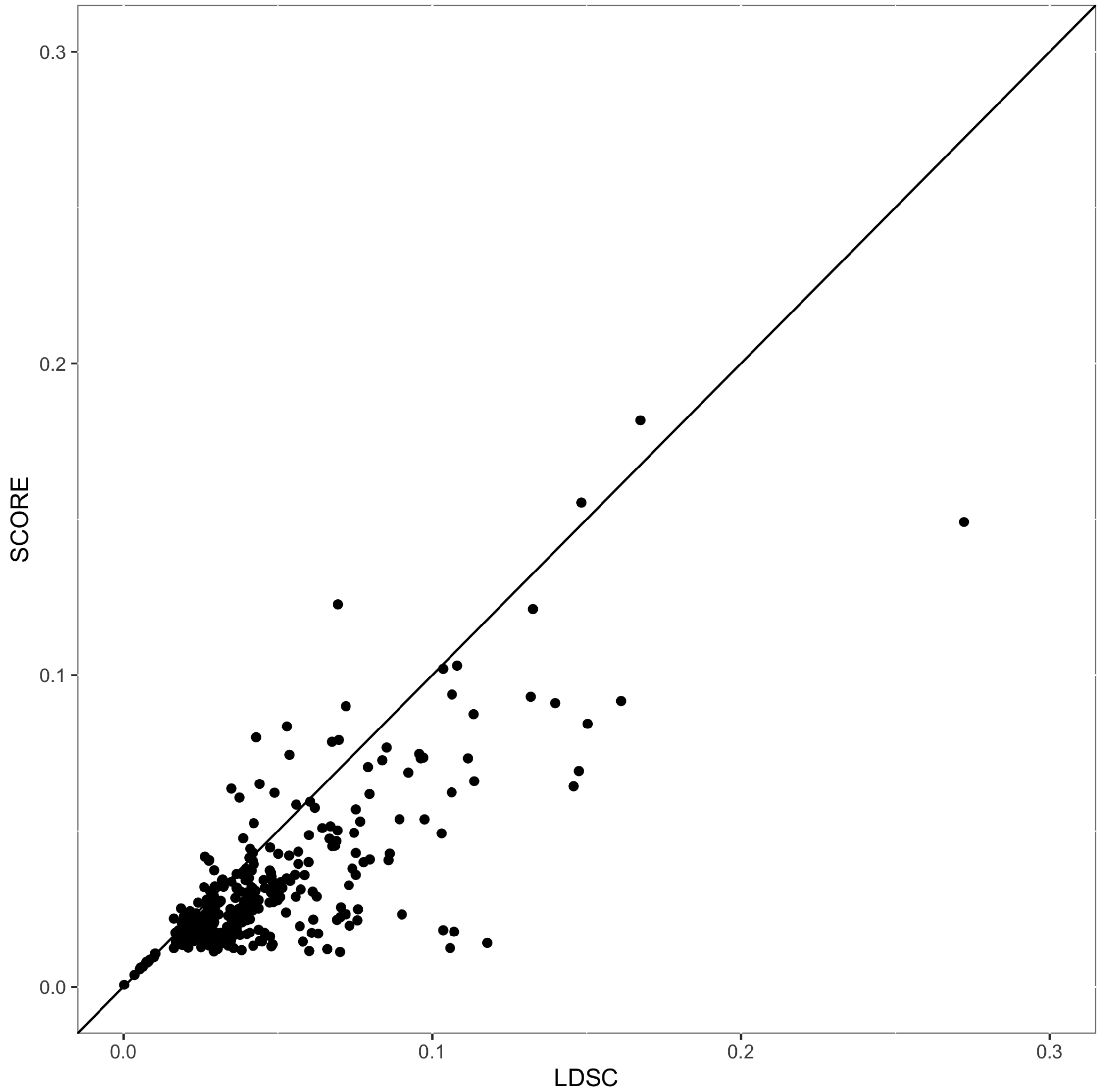

Figure S5: **Standard error estimates of genetic correlation between 28 UK biobank phenotypes with LDSC and SCORE corresponding to Figure 3.** Each dot denotes the standard error estimates of SCORE and LDSC for a pair of traits.

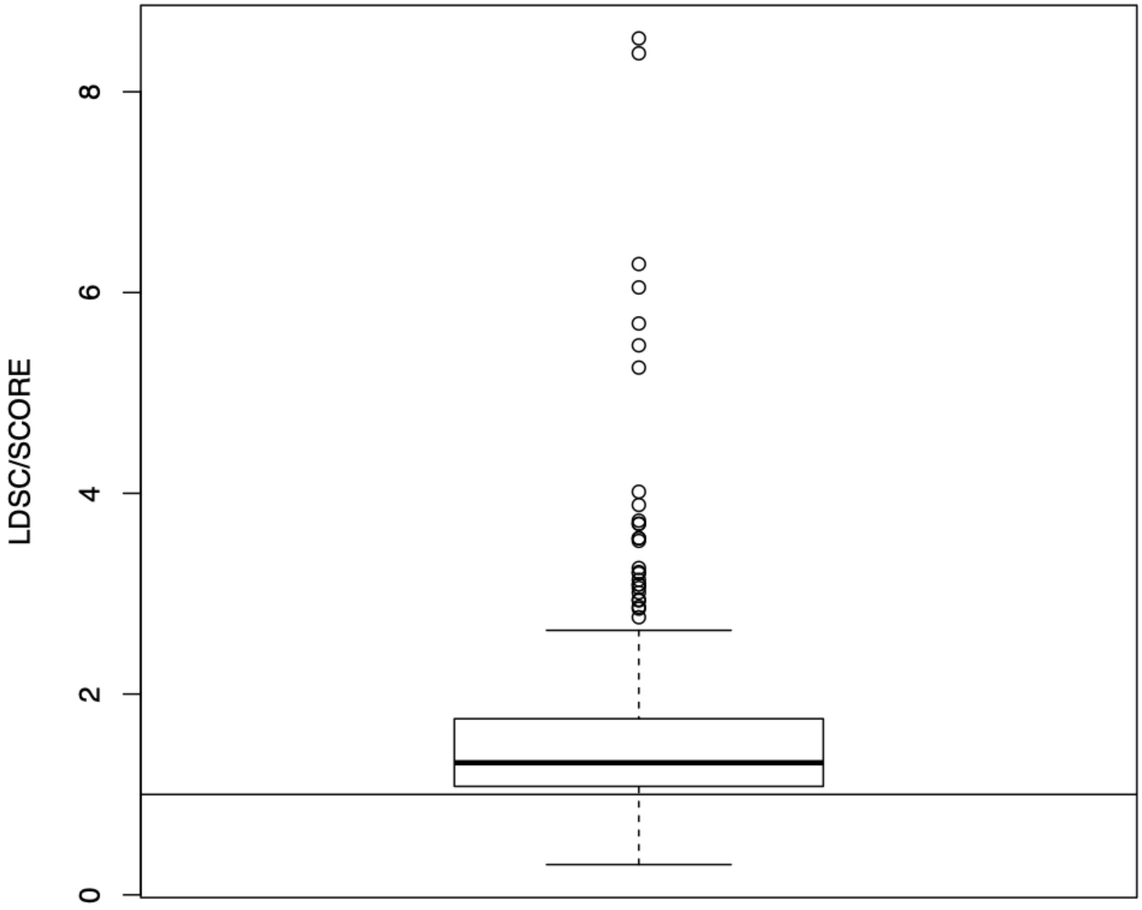

Figure S6: **Ratio of standard error estimates of genetic correlation between 28 UK biobank phenotypes with LDSC and SCORE corresponding to Figure 3.** On average, the standard error estimate of LDSC is about 1.57 times that of SCORE.

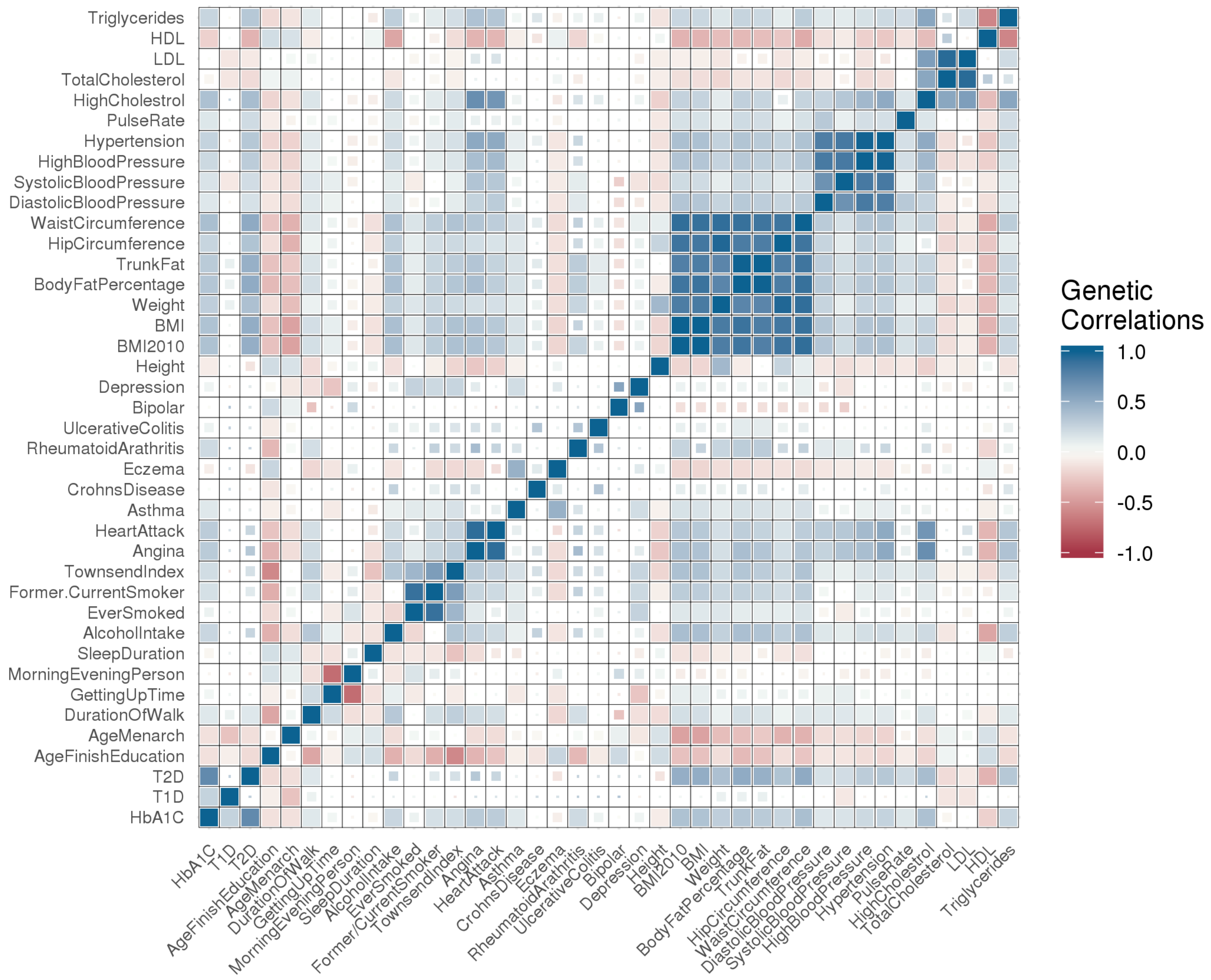

Figure S7: **Genetic correlation estimates in the UK Biobank on array SNPs**: We plot the genetic correlation estimates from SCORE across pairs of 40 phenotypes. The full list of traits and category is in Supplementary Table (Table S10). Large squares correspond to pairs that are estimated to have a statistically significant genetic correlation after Bonferroni correction at a 5% significance level while small squares indicate pairs that are significant at a 5% significance level but do not pass multiple-testing correction (see section on Quality control of UK Biobank data in Materials and Methods).

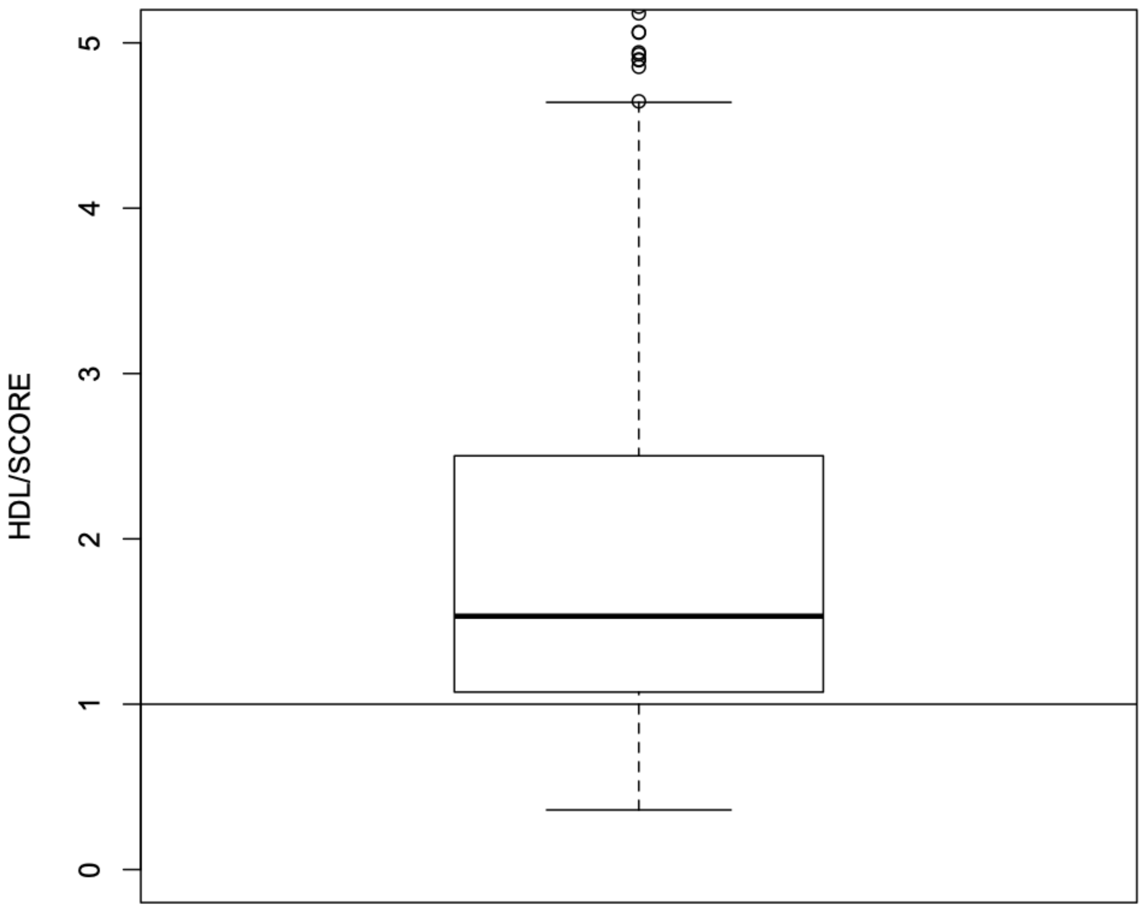

Figure S8: **Ratio of standard error estimates of genetic correlation between 40 UK biobank phenotypes with HDL and SCORE corresponding to Table S10**. The standard error estimate of HDL is about 2.53 times that of SCORE on average (median of ratio is about 1.53).

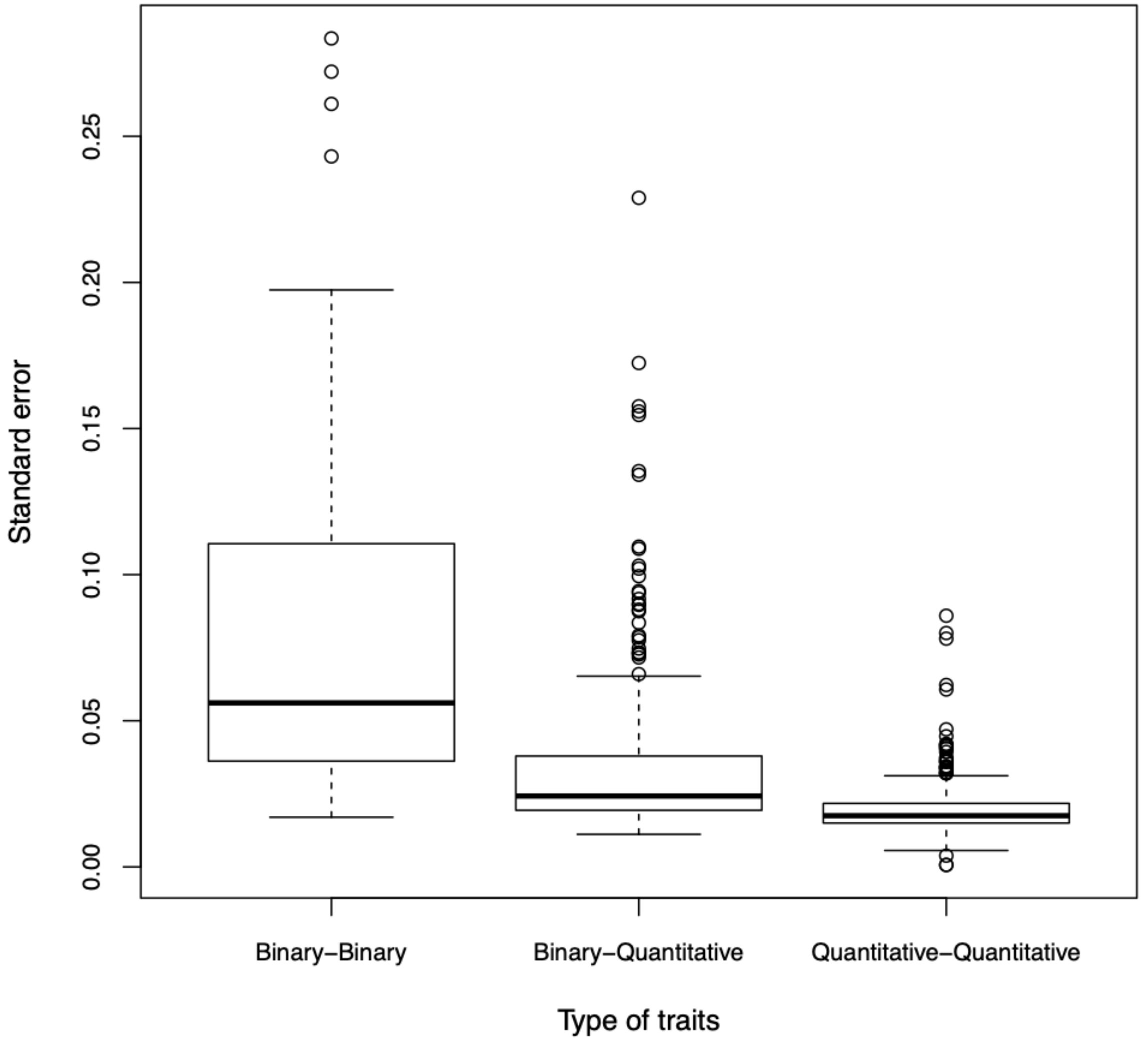

Figure S9: **Standard error of genetic correlation estimates from SCORE stratified by the type of phenotype pairs**.

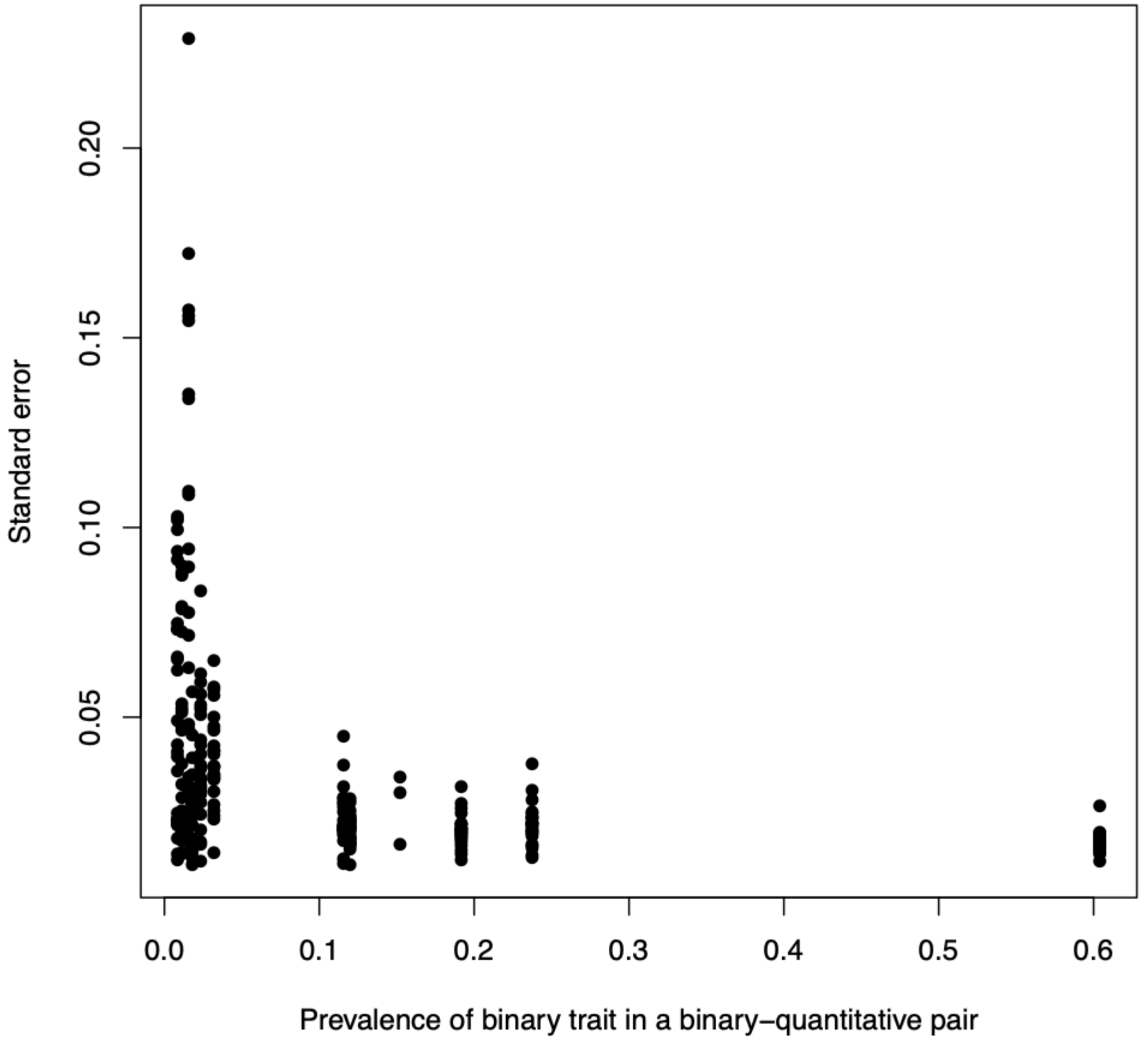

Figure S10: **Standard error of genetic correlation estimates from SCORE as a function of the prevalence of the binary phenotype when applied to a pair of phenotypes where one of traits in the pair is binary**.

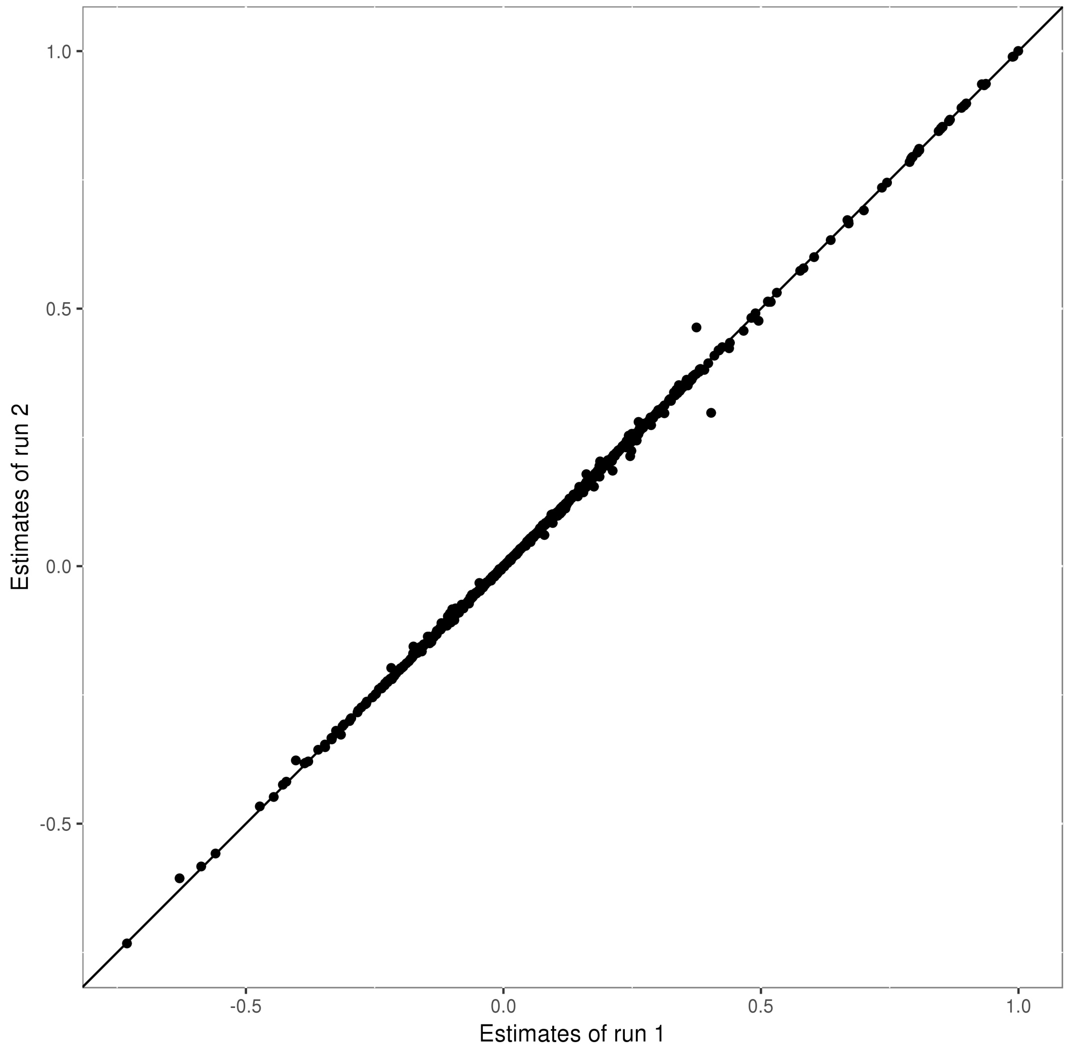

Figure S11: **Estimates of genetic correlation in the UK Biobank with different random vectors**. We plot the point estimates of genetic correlation across all pairs of 40 traits in Table S10 using SCORE with different sets of random vectors.

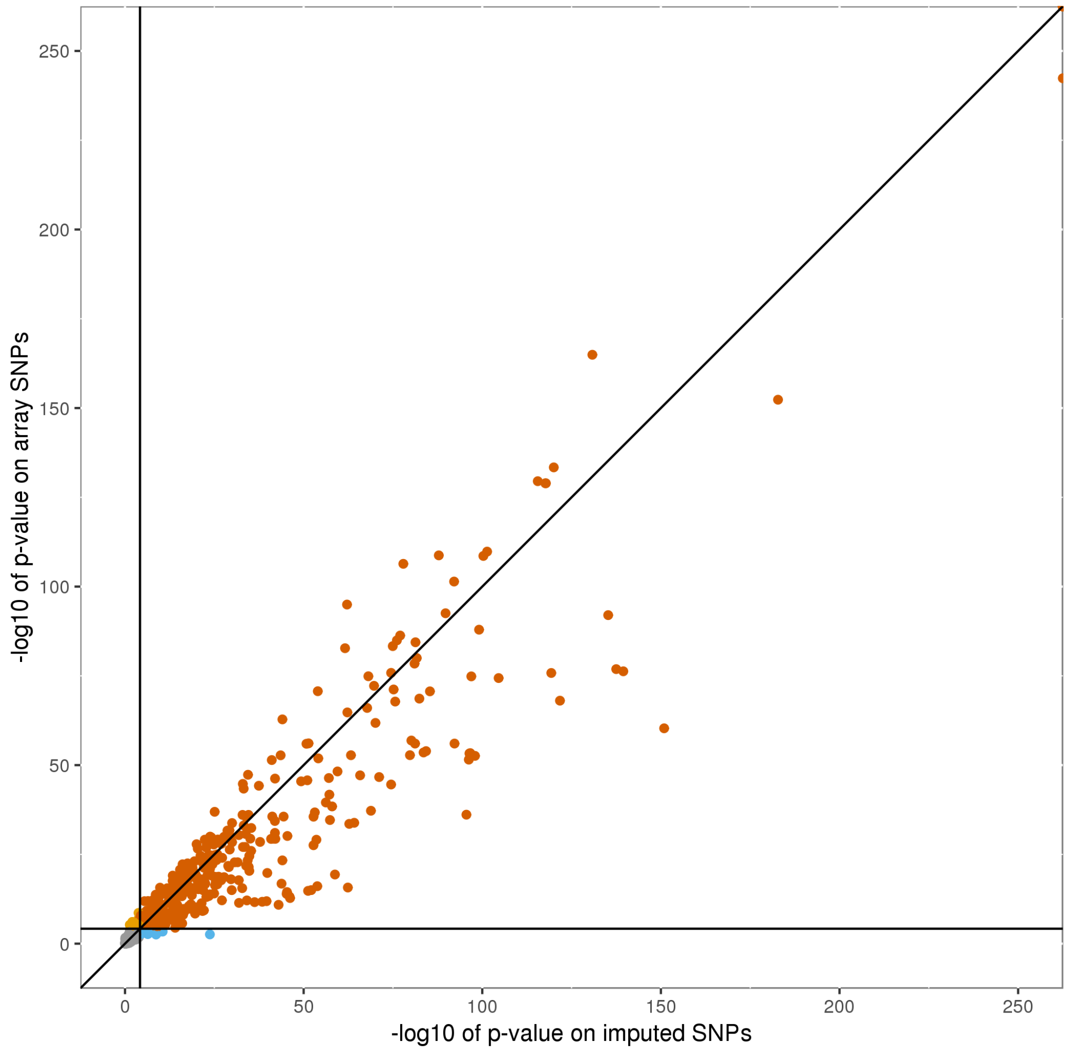

Figure S12: **Comparison of the p-values of ρ_g estimates obtained by SCORE in the UK Biobank on imputed versus array SNPs**. We analyze all pairs of 40 traits in Table S10 with SCORE on both array and imputed SNPs. We plot the $-\log_{10}$ of the p-values of a test of null hypothesis of zero genetic correlation using the two sets of SNPs. The vertical and horizontal solid lines denote the threshold of significance after Bonferroni correction, which is $-log_{10}(0.05/780)$. We found 19 pairs of significant genetic correlation unique to imputed SNPs (light orange), and 22 pairs unique to array SNPs (light blue). There are 423 pairs found significant on both sets of SNPs (dark orange) (see section on Quality control of UK Biobank data in Materials and Methods).

Supplemental Tables

Polygenicity	Method SCORE	Genetic correlation ρ_g	Bias	MSE	SE	Bias	MSE	SE	Bias	MSE	SE	Bias	MSE	SE
			$h_1^2 = 0.1, h_2^2 = 0.2$			$h_1^2 = 0.2, h_2^2 = 0.6$			$h_1^2 = 0.5, h_2^2 = 0.5$			$h_1^2 = 0.6, h_2^2 = 0.8$		
Infinitesimal	OVERLAP	0	-0.0826	0.1019	0.3083	-0.0302	0.0428	0.2048	0.0099	0.0145	0.1201	0.0083	0.0084	0.0912
	B=10	0	-0.0826	0.1018	0.3082	-0.0301	0.0428	0.2047	0.0099	0.0145	0.1201	0.0083	0.0084	0.0912
	B=100	0	-0.0826	0.1018	0.3082	-0.0301	0.0428	0.2047	0.0099	0.0145	0.1201	0.0083	0.0084	0.0912
	OVERLAP	0.2	-0.05	0.132	0.3598	-0.01	0.033	0.1814	0.0147	0.0159	0.1253	-0.0173	0.0089	0.0928
	B=10	0.2	-0.05	0.1319	0.3597	-0.01	0.033	0.1813	0.0147	0.0159	0.1253	-0.0173	0.0089	0.0928
	B=100	0.2	-0.05	0.1319	0.3597	-0.01	0.033	0.1813	0.0147	0.0159	0.1253	-0.0173	0.0089	0.0928
	OVERLAP	0.5	-0.1567	0.0841	0.244	-0.016	0.0266	0.1622	0.0115	0.0137	0.1164	0.0143	0.0047	0.0669
	B=10	0.5	-0.1568	0.0841	0.244	-0.0161	0.0266	0.1621	0.0114	0.0137	0.1164	0.0142	0.0047	0.0669
	B=100	0.5	-0.1568	0.0841	0.244	-0.0161	0.0266	0.1621	0.0116	0.0138	0.1169	0.0142	0.0047	0.0669
	OVERLAP	0.8	-0.3187	0.156	0.2334	-0.1106	0.0309	0.1365	-0.0206	0.0067	0.0789	-0.0016	0.0034	0.0583
	B=10	0.8	-0.3187	0.156	0.2333	-0.1107	0.0309	0.1365	-0.0207	0.0067	0.0789	-0.0017	0.0034	0.0583
	B=100	0.8	-0.3187	0.156	0.2333	-0.1107	0.0309	0.1365	-0.0207	0.0067	0.0789	-0.0017	0.0034	0.0583
Medium polygenicity	OVERLAP	0	0.0506	0.1227	0.3466	0.0023	0.0349	0.1869	0.0022	0.0198	0.1408	0.0052	0.0091	0.0952
	B=10	0	0.0506	0.1226	0.3465	0.0023	0.0349	0.1868	0.0022	0.0198	0.1408	0.0052	0.0091	0.0952
	B=100	0	0.0506	0.1226	0.3465	0.0023	0.0349	0.1868	0.0022	0.0198	0.1408	0.0052	0.0091	0.0952
	OVERLAP	0.2	0.0259	0.0965	0.3096	0.0116	0.0382	0.1952	-6e-04	0.0161	0.1267	-0.01	0.0065	0.0797
	B=10	0.2	0.0259	0.0965	0.3096	0.0115	0.0382	0.1952	-7e-04	0.0161	0.1267	-0.01	0.0065	0.0797
	B=100	0.2	0.0259	0.0965	0.3096	0.0115	0.0382	0.1952	-7e-04	0.0161	0.1267	-0.01	0.0065	0.0797
	OVERLAP	0.5	0.0317	0.0974	0.3104	0.0039	0.0287	0.1693	0.0197	0.0118	0.1066	0.0076	0.0083	0.0909
	B=10	0.5	0.0316	0.0973	0.3104	0.0039	0.0287	0.1693	0.0197	0.0117	0.1066	0.0076	0.0083	0.0909
	B=100	0.5	0.0316	0.0973	0.3104	0.0039	0.0287	0.1693	0.022	0.0114	0.1045	0.0076	0.0083	0.0909
	OVERLAP	0.8	-0.1181	0.1209	0.3271	0.0116	0.031	0.1758	0.0228	0.013	0.1119	0.031	0.0121	0.1054
	B=10	0.8	-0.1182	0.1209	0.327	0.0115	0.031	0.1758	0.0228	0.013	0.1118	0.0309	0.0121	0.1054
	B=100	0.8	-0.1182	0.1209	0.327	0.0115	0.031	0.1758	0.0228	0.013	0.1118	0.0309	0.0121	0.1054
Low polygenicity	OVERLAP	0	0.0694	0.136	0.3623	0.0115	0.0452	0.2123	-0.0223	0.0158	0.1236	-0.0091	0.0101	0.0999
	B=10	0	0.0694	0.136	0.3622	0.0115	0.0452	0.2123	-0.0223	0.0158	0.1236	-0.0091	0.0101	0.0999
	B=100	0	0.0694	0.136	0.3622	0.0115	0.0452	0.2123	-0.0223	0.0158	0.1236	-0.0091	0.0101	0.0999
	OVERLAP	0.2	0.0135	0.0594	0.2433	0.0366	0.0424	0.2025	0.018	0.0188	0.136	0.0193	0.0105	0.1004
	B=10	0.2	0.0135	0.0594	0.2433	0.0366	0.0424	0.2025	0.018	0.0188	0.136	0.0193	0.0105	0.1004
	B=100	0.2	0.0135	0.0594	0.2433	0.0366	0.0424	0.2025	0.018	0.0188	0.136	0.0193	0.0105	0.1004
	OVERLAP	0.5	0.0463	0.0992	0.3116	0.0478	0.0261	0.1544	0.0882	0.0215	0.1171	0.0334	0.0121	0.1048
	B=10	0.5	0.0463	0.0992	0.3116	0.0478	0.0261	0.1544	0.0882	0.0215	0.1171	0.0334	0.0121	0.1048
	B=100	0.5	0.0463	0.0992	0.3116	0.0478	0.0261	0.1544	0.0882	0.0215	0.1171	0.0334	0.0121	0.1048
	OVERLAP	0.8	0.0363	0.083	0.2857	0.0953	0.042	0.1815	0.0839	0.0253	0.1353	0.0869	0.0175	0.0998
	B=10	0.8	0.0363	0.083	0.2857	0.0953	0.042	0.1815	0.0839	0.0253	0.1353	0.0869	0.0175	0.0998
	B=100	0.8	0.0363	0.083	0.2857	0.0953	0.042	0.1815	0.0839	0.0253	0.1353	0.0869	0.0175	0.0998

Table S1: **Estimates of bias, mean square error and standard error of SCORE for varying number of random vectors $B = 10$, $B = 100$ and SCORE-OVERLAP**. See section on Simulations to assess accuracy in Materials and Methods for details.

Polygenicity	Method	Genetic correlation ρ_g	(h_1^2, h_2^2)			
			$(0.1, 0.2)$	$(0.2, 0.6)$	$(0.5, 0.5)$	$(0.6, 0.8)$
Infinitesimal	LDSC/SCORE	0	1.79	1.79	2.41	2.32
	HDL/SCORE	0	1.35	1.2	1.42	1.35
	LDSC/SCORE	0.2	1.26	1.64	2.63	2.32
	HDL /SCORE	0.2	1.05	1.48	1.47	1.5
	LDSC/SCORE	0.5	1.69	2	2.19	2.6
	HDL/SCORE	0.5	1.65	1.14	1.18	1.39
	LDSC/SCORE	0.8	1.4	1.62	1.95	2.16
	HDL/SCORE	0.8	1.26	1.23	1.41	1.28
Medium polygenicity	LDSC/SCORE	0	1.4	1.96	2.08	2.07
	HDL/SCORE	0	1.25	1.41	1.21	1.36
	LDSC/SCORE	0.2	1.74	2.09	2.15	2.28
	HDL/SCORE	0.2	1.44	1.54	1.38	1.4
	LDSC/SCORE	0.5	1.49	1.96	2.59	2.37
	HDL/SCORE	0.5	1.41	1.32	1.54	1.42
	LDSC/SCORE	0.8	1.6	2.01	2.42	2.2
	HDL/SCORE	0.8	1.17	1.45	1.61	1.23
Low polygenicity	LDSC/SCORE	0	1.08	1.92	2.57	2.17
	HDL/SCORE	0	1.11	1.18	1.27	1.15
	LDSC/SCORE	0.2	1.79	1.57	1.97	2.24
	HDL/SCORE	0.2	1.31	1.07	1.29	1.3
	LDSC/SCORE	0.5	1.18	2.25	2.49	1.9
	HDL/SCORE	0.5	1.08	1.44	1.26	1.29
	LDSC/SCORE	0.8	1.45	1.81	2.47	2.37
	HDL/SCORE	0.8	1.24	1.28	1.38	1.36

Table S2: **Ratio of SE of summary-statistic methods relative to SCORE ($N = 5,000$ individuals, $M = 305,630$ SNPs).** See section on Simulations to assess accuracy in Materials and Methods for details.

Method	Genetic correlation ρ_g	Bias	MSE	SE	Bias	MSE	SE	Bias	MSE	SE	Bias	MSE	SE
		$h_1^2 = 0.1, h_2^2 = 0.2$			$h_1^2 = 0.2, h_2^2 = 0.6$			$h_1^2 = 0.5, h_2^2 = 0.5$			$h_1^2 = 0.6, h_2^2 = 0.8$		
GCTA-GREML	0	-0.0818	0.0998	0.305	-0.029	0.0395	0.197	0.0096	0.013	0.114	0.0014	0.0076	0.0871
GCTA-HE	0	-0.104	0.217	0.454	0.0366	0.0878	0.294	0.0026	0.0268	0.164	0.0225	0.0148	0.119
HDL	0	-0.0265	0.172	0.414	-0.0095	0.0606	0.246	0.0185	0.0294	0.17	0.007	0.0153	0.123
LDSC	0	0.0519	0.3055	0.5503	-0.007	0.1343	0.3665	-0.0014	0.0837	0.2894	0.0083	0.045	0.2119
SCORE	0	-0.0826	0.102	0.308	-0.0302	0.0428	0.205	0.0099	0.0145	0.12	0.0083	0.0084	0.0912
GCTA-GREML	0.2	-0.0571	0.123	0.346	-0.0103	0.0328	0.181	0.0074	0.0152	0.123	-0.0114	0.0077	0.0868
GCTA-HE	0.2	-0.188	0.261	0.475	-0.0234	0.0554	0.234	0.029	0.0321	0.177	-0.0263	0.0157	0.123
HDL	0.2	-0.094	0.151	0.377	0.0254	0.0729	0.269	0.0236	0.0346	0.184	0.0027	0.0194	0.139
LDSC	0.2	-0.0946	0.2134	0.4522	0.0134	0.0885	0.2973	0.0177	0.1088	0.3293	0.0051	0.0463	0.215
SCORE	0.2	-0.05	0.132	0.36	-0.01	0.033	0.181	0.0147	0.0159	0.125	-0.0173	0.0089	0.0928
GCTA-GREML	0.5	-0.158	0.0858	0.247	-0.012	0.0244	0.156	0.0159	0.0121	0.109	0.0092	0.0045	0.0662
GCTA-HE	0.5	-0.142	0.161	0.375	-0.0394	0.0593	0.24	0.0181	0.0224	0.149	0.0111	0.0116	0.107
HDL	0.5	-0.162	0.188	0.401	-0.0195	0.0345	0.185	0.0047	0.0189	0.138	0.0207	0.009	0.0928
LDSC	0.5	-0.3298	0.278	0.4114	-0.0955	0.1143	0.3243	0	0.0649	0.2547	0.0308	0.0312	0.1739
SCORE	0.5	-0.1567	0.0841	0.244	-0.016	0.0266	0.1622	0.0115	0.0137	0.1164	0.0143	0.0047	0.0669
GCTA-GREML	0.8	-0.317	0.156	0.235	-0.106	0.0283	0.131	-0.0232	0.0073	0.082	-0.0004	0.0033	0.0571
GCTA-HE	0.8	-0.389	0.287	0.369	-0.115	0.0446	0.177	-0.023	0.0139	0.116	0.0093	0.0061	0.0778
HDL	0.8	-0.391	0.239	0.294	-0.154	0.0518	0.168	-0.0421	0.0141	0.111	-0.0114	0.0057	0.0748
LDSC	0.8	-0.4841	0.3415	0.3273	-0.1851	0.0833	0.2215	-0.0727	0.029	0.1541	-0.019	0.0162	0.1257
SCORE	0.8	-0.319	0.156	0.233	-0.111	0.0309	0.137	-0.0206	0.0067	0.0789	-0.0016	0.0034	0.0583

Table S3: **Bias, mean square error and standard error of genetic correlation estimation methods in simulations corresponding to Figure S1 ($N = 5,000$ individuals, $M = 305,630$ SNPs).**

Method	Genetic correlation ρ_g	Bias	MSE	SE	Bias	MSE	SE	Bias	MSE	SE	Bias	MSE	SE
		$h_1^2 = 0.1, h_2^2 = 0.2$			$h_1^2 = 0.2, h_2^2 = 0.6$			$h_1^2 = 0.5, h_2^2 = 0.5$			$h_1^2 = 0.6, h_2^2 = 0.8$		
GCTA-GREML	0	0.0414	0.12	0.344	0.003	0.0343	0.185	-0.0025	0.0157	0.125	-0.0011	0.0075	0.0866
GCTA-HE	0	0.0964	0.243	0.484	0.0163	0.0502	0.223	0.0103	0.0328	0.181	-0.0051	0.0159	0.126
HDL	0	0.0626	0.193	0.434	-0.0225	0.0699	0.263	0.0103	0.0292	0.171	0.0155	0.0171	0.13
LDSC	0	0.0608	0.2375	0.4835	-0.0273	0.1344	0.3656	0.0179	0.0862	0.2931	0.0301	0.0399	0.1975
SCORE	0	0.0506	0.123	0.347	0.0023	0.0349	0.187	0.0022	0.0198	0.141	0.0052	0.0091	0.0952
GCTA-GREML	0.2	-0.0034	0.12	0.347	0.0143	0.0376	0.193	-0.0055	0.0159	0.126	-0.007	0.0058	0.0759
GCTA-HE	0.2	0.0162	0.2	0.447	0.0182	0.0706	0.265	-0.0073	0.0298	0.172	-0.0247	0.0168	0.127
HDL	0.2	-0.0066	0.198	0.445	0.0155	0.0902	0.3	0.005	0.0307	0.175	-0.0121	0.0125	0.111
LDSC	0.2	-0.0846	0.2979	0.5392	0.0209	0.1664	0.4073	-0.0089	0.0744	0.2726	0.0052	0.033	0.1815
SCORE	0.2	0.0259	0.0965	0.31	0.0116	0.0382	0.195	-0.0006	0.0161	0.127	-0.01	0.0065	0.0797
GCTA-GREML	0.5	0.0292	0.102	0.319	0.0081	0.0289	0.17	0.0182	0.0121	0.108	0.0068	0.0075	0.0861
GCTA-HE	0.5	-0.0213	0.146	0.382	0.0061	0.0576	0.24	0.0138	0.0226	0.15	0.0155	0.0155	0.123
HDL	0.5	0.0137	0.124	0.352	-0.0255	0.0552	0.234	0.0223	0.0304	0.173	0.0055	0.0167	0.129
LDSC	0.5	-0.1026	0.2247	0.4628	-0.0634	0.1138	0.3313	-0.0142	0.0766	0.2764	-0.0202	0.0467	0.2151
SCORE	0.5	0.0317	0.0974	0.31	0.0039	0.0287	0.169	0.0197	0.0118	0.107	0.0076	0.0083	0.0909
GCTA-GREML	0.8	-0.112	0.12	0.328	0.0146	0.0301	0.173	0.0164	0.0134	0.115	0.036	0.0095	0.0904
GCTA-HE	0.8	-0.0941	0.212	0.451	0.0307	0.0454	0.211	0.0151	0.0277	0.166	0.0288	0.0185	0.133
HDL	0.8	-0.155	0.172	0.385	0.0355	0.0665	0.255	0.0362	0.0336	0.18	0.0453	0.019	0.13
LDSC	0.8	-0.2322	0.3293	0.5248	0.0022	0.125	0.3536	0.0144	0.0733	0.2704	0.0134	0.0542	0.2324
SCORE	0.8	-0.118	0.121	0.327	0.0116	0.031	0.176	0.0228	0.013	0.112	0.031	0.0121	0.105

Table S4: **Bias, mean square error and standard error of genetic correlation estimation methods in simulations corresponding to Figure S2 ($N = 5,000$ individuals, $M = 305,630$ SNPs).**

Method	Genetic correlation ρ_g	Bias	MSE	SE	Bias	MSE	SE	Bias	MSE	SE	Bias	MSE	SE
		$h_1^2 = 0.1, h_2^2 = 0.2$			$h_1^2 = 0.2, h_2^2 = 0.6$			$h_1^2 = 0.5, h_2^2 = 0.5$			$h_1^2 = 0.6, h_2^2 = 0.8$		
GCTA-GREML	0	0.0396	0.1156	0.3377	0.0188	0.0339	0.183	-0.0248	0.0153	0.1213	-0.0084	0.0084	0.0912
GCTA-HE	0	-0.0011	0.1867	0.4321	0.0314	0.0715	0.2656	-0.02	0.028	0.1662	-0.0041	0.0138	0.1174
HDL	0	0.0286	0.1395	0.3724	0.0212	0.0635	0.2511	-0.007	0.0245	0.1565	-0.0058	0.0132	0.1145
LDSC	0	-0.0844	0.1544	0.3838	0.0206	0.1595	0.3989	0.0035	0.1012	0.3181	0.0082	0.0471	0.2168
SCORE	0	0.0694	0.136	0.3623	0.0115	0.0452	0.2123	-0.0223	0.0158	0.1236	-0.0091	0.0101	0.0999
GCTA-GREML	0.2	-0.0198	0.0722	0.268	0.0296	0.031	0.1736	0.014	0.0167	0.1285	0.0115	0.0089	0.0936
GCTA-HE	0.2	0.0051	0.1576	0.3969	0.0224	0.0701	0.2639	0.012	0.0273	0.1647	0.0315	0.0165	0.1245
HDL	0.2	0.0812	0.1502	0.379	0.0744	0.0527	0.2173	0.0054	0.031	0.1761	0.0143	0.0171	0.1302
LDSC	0.2	0.0072	0.2668	0.5165	0.0589	0.105	0.3187	-0.0129	0.0719	0.2678	0.0119	0.0506	0.2246
SCORE	0.2	0.0135	0.0594	0.2433	0.0366	0.0424	0.2025	0.018	0.0188	0.136	0.0193	0.0105	0.1004
GCTA-GREML	0.5	0.0353	0.1053	0.3226	0.0472	0.0206	0.1356	0.0893	0.0206	0.1122	0.0379	0.0102	0.0936
GCTA-HE	0.5	0.0019	0.1984	0.4454	0.0411	0.0584	0.2382	0.1056	0.0352	0.1552	0.0527	0.021	0.1352
HDL	0.5	0.0171	0.1102	0.3315	0.0397	0.0537	0.2284	0.1039	0.0327	0.1481	0.0362	0.0195	0.1348
LDSC	0.5	0.1266	0.1518	0.3684	0.0636	0.1265	0.3499	0.0994	0.0952	0.2921	0.0514	0.0423	0.1992
SCORE	0.5	0.0463	0.0992	0.3116	0.0478	0.0261	0.1544	0.0882	0.0215	0.1171	0.0334	0.0121	0.1048
GCTA-GREML	0.8	0.0725	0.1016	0.3103	0.0939	0.0411	0.1796	0.0814	0.0235	0.1297	0.0876	0.0156	0.0888
GCTA-HE	0.8	-0.0097	0.1859	0.431	0.0844	0.0649	0.2405	0.0806	0.0393	0.1811	0.0765	0.0189	0.1144
HDL	0.8	0.1205	0.144	0.3598	0.0922	0.0625	0.2324	0.0993	0.0447	0.1867	0.0888	0.0262	0.1353
LDSC	0.8	0.0414	0.1724	0.4132	0.1331	0.1256	0.3284	0.0853	0.1186	0.3337	0.0741	0.0614	0.2365
SCORE	0.8	0.0363	0.083	0.2857	0.0953	0.042	0.1815	0.0839	0.0253	0.1353	0.0869	0.0175	0.0998

Table S5: **Bias, mean square error and standard error of genetic correlation estimation methods in simulations corresponding to Fig S3 ($N = 5,000$ individuals, $M = 305,630$ SNPs).**

Method	Overlap Proportion	Bias	MSE	SE
LDSC	0	-0.1672	0.1405	0.3355
	0.2	0.0044	0.0921	0.3035
	0.5	0.1708	0.1022	0.2702
	0.8	0.0808	0.0815	0.2737
	1	-0.0955	0.1143	0.3243
SCORE	0	-0.0037	0.0405	0.2013
	0.2	0.2242	0.0753	0.1582
	0.5	0.2843	0.0916	0.1036
	0.8	0.2722	0.087	0.1428
	1	-0.016	0.0266	0.1622
GCTA-GREML	0	-0.0063	0.0383	0.1956
	0.2	0.2635	0.0957	0.1619
	0.5	0.3301	0.1219	0.1137
	0.8	0.2371	0.0788	0.1505
	1	-0.012	0.0244	0.1558

Table S6: **Accuracy of SCORE, LDSC, and GCTA-GREML as a function of varying sample overlap corresponding to Figure S4**. See section on Simulations to assess impact of sample overlap in Materials and Methods for details.

Polygenicity	(h_1^2, h_2^2)	ρ_g	$\hat{SE}$	SE
Infinitesimal	0.1, 0.2	0	0.45	0.42
	0.2, 0.6	0	0.2	0.2
	0.5, 0.5	0	0.12	0.12
	0.6, 0.8	0	0.09	0.09
	0.1, 0.2	0.2	0.4	0.38
	0.2, 0.6	0.2	0.19	0.19
	0.5, 0.5	0.2	0.12	0.12
	0.6, 0.8	0.2	0.09	0.09
	0.1, 0.2	0.5	0.41	0.3
	0.2, 0.6	0.5	0.18	0.17
	0.5, 0.5	0.5	0.11	0.11
	0.6, 0.8	0.5	0.07	0.07
	0.1, 0.2	0.8	0.41	0.34
	0.2, 0.6	0.8	0.18	0.15
	0.5, 0.5	0.8	0.09	0.09
	0.6, 0.8	0.8	0.05	0.05
Medium polygenicity	0.1, 0.2	0	0.45	0.39
	0.2, 0.6	0	0.19	0.19
	0.5, 0.5	0	0.12	0.14
	0.6, 0.8	0	0.09	0.09
	0.1, 0.2	0.2	0.43	0.39
	0.2, 0.6	0.2	0.18	0.19
	0.5, 0.5	0.2	0.12	0.12
	0.6, 0.8	0.2	0.09	0.08
	0.1, 0.2	0.5	0.43	0.36
	0.2, 0.6	0.5	0.18	0.18
	0.5, 0.5	0.5	0.12	0.11
	0.6, 0.8	0.5	0.09	0.09
	0.1, 0.2	0.8	0.44	0.36
	0.2, 0.6	0.8	0.20	0.18
	0.5, 0.5	0.8	0.12	0.11
	0.6, 0.8	0.8	0.1	0.11
Low polygenicity	0.1, 0.2	0	0.39	0.38
	0.2, 0.6	0	0.21	0.2
	0.5, 0.5	0	0.12	0.12
	0.6, 0.8	0	0.1	0.09
	0.1, 0.2	0.2	0.35	0.29
	0.2, 0.6	0.2	0.19	0.2
	0.5, 0.5	0.2	0.12	0.14
	0.6, 0.8	0.2	0.19	0.2
	0.1, 0.2	0.5	0.42	0.33
	0.2, 0.6	0.5	0.18	0.16
	0.5, 0.5	0.5	0.13	0.12
	0.6, 0.8	0.5	0.09	0.1
	0.1, 0.2	0.8	0.35	0.3
	0.2, 0.6	0.8	0.19	0.18
	0.5, 0.5	0.8	0.12	0.12
	0.6, 0.8	0.8	0.1	0.1

Table S7: **Assessment of Jackknife estimates of standard error ($N = 5,000$ samples and $305,630$ SNPs, block size = $4,000$ SNPs).** We report the average of estimates of standard error across 100 replicates.

Small-scale simulations		
Polygenicity	(h_1^2, h_2^2)	FPR
Infinitesimal	0.1, 0.2	0.014
	0.2 ,0.6	0.037
	0.5, 0.5	0.01
	0.6, 0.8	0.07
Medium polygenicity	0.1, 0.2	0
	0.2, 0.6	0.026
	0.5, 0.5	0.071
	0.6, 0.8	0.02
Low polygenicity	0.1, 0.2	0.065
	0.2, 0.6	0.054
	0.5, 0.5	0.05
	0.6, 0.8	0.06
Large-scale simulations		
Prevelance	(h_1^2, h_2^2)	FPR
Continuous	0.272, 0.12	0.04
50%	0.272, 0.12	0.08
10%	0.272, 0.12	0.07
1%	0.272, 0.12	0.08
0.5%	0.272, 0.12	0.02
0.01%	0.272, 0.12	0

Table S8: **The false positive rate of SCORE is controlled**. We evaluated the false positive rate of SCORE in simulations where ρ_g is zero. We considered small-scale ($N = 5,000$ individuals and $M = 305,630$ SNPs) and large-scale simulations ($N = 291,273$ individuals and $M = 305,630$ SNPs). We also considered simulations with binary traits with varying prevalence. Standard error esimates were obtained using a Block Jackknife with a block size of 4000 SNPs. For each genetic architecture, we performed 100 replicates and reported the FPR as the rate with which SCORE rejects the null hypothesis of $\rho_g = 0$.

h_1^2	h_2^2	ρ_g	ρ_e	Prevalence	$\overline{\rho_g}$	SE	p-value
0.272	0.12	0	0	Continuous trait	0.001	0.018	0.53
0.272	0.12	-0.23	0	Continuous trait	-0.238	0.093	0.39
0.272	0.12	-0.23	0	50%	-0.238	0.097	0.41
0.272	0.12	-0.23	0	25%	-0.239	0.101	0.43
0.272	0.12	-0.23	0	10%	-0.238	0.103	0.44
0.272	0.12	-0.23	0	1%	-0.234	0.124	0.75
0.272	0.12	-0.23	0	0.5%	-0.248	0.134	0.18
0.272	0.12	-0.23	0	0.01%	-0.215	0.205	0.44
0.272	0.12	-0.23	-0.04	Continuous trait	-0.211	0.107	0.08
0.272	0.12	-0.23	-0.04	0.01%	-0.243	0.342	0.70
0.272	0.12	-0.23	0.04	Continuous trait	-0.221	0.089	0.52
0.272	0.12	-0.23	0.04	0.01%	-0.235	0.352	0.89

Table S9: **Estimates of ρ_g as a function of the prevalence of binary traits ($N = 291,273$ individuals and $305,630$ SNPs).** We report the average of the point estimates of ρ_g, the SE and p-value of a test of the null hypothesis that the estimates of ρ_g are unbiased. We compute p-values of a test of no bias from the Z-score defined as $\frac{\overline{\rho_g}}{SE/10}$. See section on simulations to assess accuracy for binary traits in Materials and Methods for details.

Lipid metabolism traits	Triglycerides
Lipid metabolism traits	HDL
Lipid metabolism traits	LDL
Lipid metabolism traits	Total Cholesterol
Lipid metabolism traits	High Cholesterol
Blood pressure and circulatory traits	Pulse Rate
Blood pressure and circulatory traits	Hypertension
Blood pressure and circulatory traits	High Blood Pressure
Blood pressure and circulatory traits	Systolic Blood Pressure
Blood pressure and circulatory traits	Diastolic Blood Pressure
Anthropometric traits	Waist Circumference
Anthropometric traits	Hip Circumference
Anthropometric traits	Trunk Fat
Anthropometric traits	Body Fat Percentage
Anthropometric traits	Weight
Anthropometric traits	BMI
Anthropometric traits	BMI2010
Anthropometric traits	Height
Psychiatric disorders	Depression
Psychiatric disorders	Bipolar
Autoimmune disorders	Ulcerative Colitis
Autoimmune disorders	Rheumatoid Arthritis
Autoimmune disorders	Eczema
Autoimmune disorders	Crohn’s Disease
Autoimmune disorders	Asthma
Coronary artery disease related traits	Heart Attack
Coronary artery disease related traits	Angina
Environmental factor traits	Townsend Index
Environmental factor traits	Former/Current Smoker
Environmental factor traits	Ever Smoked
Environmental factor traits	Alcohol Intake
Socioeconomic and general medical information traits	Sleep Duration
Socioeconomic and general medical information traits	Morning Evening Person
Socioeconomic and general medical information traits	Getting Up Time
Socioeconomic and general medical information traits	Duration Of Walk
Socioeconomic and general medical information traits	Age Menarch
Socioeconomic and general medical information traits	Age Finish Education
Glucose metabolism and diabetes traits	T2D
Glucose metabolism and diabetes traits	T1D
Glucose metabolism and diabetes traits	HbA1C

Table S10: **Forty traits in the UK Biobank analyzed in this study**

Supplemental Methods

SCORE-OVERLAP: Estimating genetic correlation when both traits are measured on the same set of individuals

Here we describe our model in the setting where the two traits are measured on the same set of individuals. Let $\boldsymbol{X}$ denote the genotype matrix for which both traits are observed. Denote the concatenated phenotype vector, $\boldsymbol{y} \equiv [\boldsymbol{y}_1^T, \boldsymbol{y}_2^T]^T$, concatenated environmental effect vector $\boldsymbol{\epsilon} \equiv [\boldsymbol{\epsilon_1}^T, \boldsymbol{\epsilon_2}^T]^T$, and concatenated effect size vector$\boldsymbol{\beta} \equiv [\boldsymbol{\beta_1}^T, \boldsymbol{\beta_2}^T]^T$. In this setting, the modified generative model is:

$$\boldsymbol{y} = \begin{bmatrix} \boldsymbol{X} & 0 \\ 0 & \boldsymbol{X} \end{bmatrix} \boldsymbol{\beta} + \boldsymbol{\epsilon} \tag{1}$$

In this model, the population covariance of the concatenated phenotype vector $\boldsymbol{y}$ is given by:

$$cov(\boldsymbol{y}) = \mathbb{E}[\boldsymbol{y}\boldsymbol{y}^T] - \mathbb{E}[\boldsymbol{y}]\mathbb{E}[\boldsymbol{y}]^T = \begin{bmatrix} \sigma_{g1}^2 \boldsymbol{K} & \gamma_g \boldsymbol{K} \\ \gamma_g \boldsymbol{K}^T & \sigma_{g2}^2 \boldsymbol{K} \end{bmatrix} + \begin{bmatrix} \sigma_{e1}^2 I_N & \gamma_e \boldsymbol{I}_N \\ \gamma_e I_N & \sigma_{e2}^2 \boldsymbol{I}_N \end{bmatrix} \tag{2}$$

Here $\boldsymbol{K} = \frac{\boldsymbol{X}\boldsymbol{X}^T}{M}$ is the genetic relatedness matrix (GRM). $\sigma_{gt}^2, \sigma_{et}^2$ denote the genetic and environmental variance components associated with trait t. Our approach to estimate both the variance components and the genetic correlation relies on a Method-of-Moments (MoM) estimator obtained by equating the population covariance to the empirical covariance. The empirical covariance of the concatenated phenotype vector $\boldsymbol{y}$ is estimated by the sample covariance: $\boldsymbol{y}\boldsymbol{y}^T$. The MoM estimator is obtained by solving the following ordinary least squares problem:

$$(\widehat{\gamma_g}, \widehat{\gamma_e}, \widehat{\sigma_{g1}^2}, \widehat{\sigma_{g2}^2}, \widehat{\sigma_{e1}^2}, \widehat{\sigma_{e2}^2}) = argmin_{\gamma_g,\gamma_e,\sigma_{g1}^2,\sigma_{g2}^2,\sigma_{e1}^2,\sigma_{e2}^2} ||\boldsymbol{y}\boldsymbol{y}^T - (\begin{bmatrix} \sigma_{g1}^2 \boldsymbol{K} & \gamma_g \boldsymbol{K} \\ \gamma_g \boldsymbol{K}^T & \sigma_{g2}^2 \boldsymbol{K} \end{bmatrix} + \begin{bmatrix} \sigma_{e1}^2 I_N & \gamma_e I_N \\ \gamma_e I_N & \sigma_{e2}^2 I_N \end{bmatrix})||_F^2 \tag{3}$$

Setting the gradient of the objective function to zero gives us the normal equations. We observe that solving for the genetic and environmental covariance parameters (γ_g, γ_e) is decoupled from solving for the variance component parameters: $\sigma_{g1}^2, \sigma_{e1}^2, \sigma_{g2}^2, \sigma_{e2}^2$. Thus, MoM estimates of the

covariance parameters can be obtained by solving the set of normal equations:

$$\begin{bmatrix} tr(\boldsymbol{K}^2) & tr(\boldsymbol{K}) \\ tr(\boldsymbol{K}) & N \end{bmatrix} \begin{bmatrix} \widehat{\gamma_g} \\ \widehat{\gamma_e} \end{bmatrix} = \begin{bmatrix} \boldsymbol{y}_2^T \boldsymbol{K} \boldsymbol{y}_1 \\ \boldsymbol{y}_2^T \boldsymbol{y}_1 \end{bmatrix} \tag{4}$$

The GRM $\boldsymbol{K}$ can be computed in time $\mathcal{O}(MN^2)$ and $\mathcal{O}(N^2)$ memory. Given the GRM, computing the coefficients for the normal equations requires $\mathcal{O}(N^2)$ time. Given each of the coefficients, we can solve analytically for $\widehat{\gamma_g}$, and $\widehat{\gamma_e}$:

$$\widehat{\gamma_g} = \frac{\boldsymbol{y}_1^T \boldsymbol{K} \boldsymbol{y}_2 - \boldsymbol{y}_1^T \boldsymbol{y}_2}{tr[\boldsymbol{K}^2] - N}$$

Here we have used the property that $tr(\boldsymbol{K}) = N$ due to the use of a standardized genotype matrix.

Similarly, we can solve the following linear systems for the estimators of each of the genetic variance parameters:

$$\begin{bmatrix} tr(\boldsymbol{K}^2) & tr(\boldsymbol{K}) \\ tr(\boldsymbol{K}) & N \end{bmatrix} \begin{bmatrix} \widehat{\sigma_{g1}^2} & \widehat{\sigma_{g2}^2} \\ \widehat{\sigma_{e1}^2} & \widehat{\sigma_{e2}^2} \end{bmatrix} = \begin{bmatrix} \boldsymbol{y}_1^T \boldsymbol{K} \boldsymbol{y}_1 & \boldsymbol{y}_2^T \boldsymbol{K} \boldsymbol{y}_2 \\ \boldsymbol{y}_1^T \boldsymbol{y}_1 & \boldsymbol{y}_2^T \boldsymbol{y}_2 \end{bmatrix} \tag{5}$$

The estimators for σ_{g1}^2 and σ_{g2}^2 are given by $\widehat{\sigma_{g1}^2} = \frac{\boldsymbol{y}_1^T \boldsymbol{K} \boldsymbol{y}_1 - \boldsymbol{y}_1^T \boldsymbol{y}_1}{tr[\boldsymbol{K}^2] - N}$ and $\widehat{\sigma_{g2}^2} = \frac{\boldsymbol{y}_2^T \boldsymbol{K} \boldsymbol{y}_2 - \boldsymbol{y}_2^T \boldsymbol{y}_2}{tr[\boldsymbol{K}^2] - N}$

Finally, we use estimates of the genetic variance parameters to obtain a plug-in estimate of the genetic correlation: $\widehat{\rho_g} = \frac{\widehat{\gamma_g}}{\sqrt{\widehat{\sigma_{g1}^2}}\sqrt{\widehat{\sigma_{g2}^2}}}$.

Substituting the expressions for the genetic covariance and variances and the GRM gives us the following estimator of genetic correlation:

$$\widehat{\rho_g} = \frac{\boldsymbol{y}_1^T \boldsymbol{K} \boldsymbol{y}_2 - \boldsymbol{y}_1^T \boldsymbol{y}_2}{\sqrt{\boldsymbol{y}_1^T \boldsymbol{K} \boldsymbol{y}_1 - \boldsymbol{y}_1^T \boldsymbol{y}_1}\sqrt{\boldsymbol{y}_2^T \boldsymbol{K} \boldsymbol{y}_2 - \boldsymbol{y}_2^T \boldsymbol{y}_2}} \tag{6}$$

Directly computing $\widehat{\rho_g}$ requires only computing $\boldsymbol{X}^T \boldsymbol{y}_1$, $\boldsymbol{X}^T \boldsymbol{y}_2$ and does not require computation of the GRM. Using the fact that the genotype matrix only contains entries in $\{0, 1, 2, \}$, we can compute these quantities in time $\mathcal{O}(\frac{NM}{max(\log_3 N, \log_3 M)})$ [2]. Thus, when phenotypes are measured on the same set of samples, SCORE-OVERLAP can efficiently estimate ρ_g with no randomization.

Modeling fixed-effect covariates

Let $\boldsymbol{W_1}$ and $\boldsymbol{W_2}$ denote the corresponding covariate matrices for each trait. To include covariate, the generative model in Equation 1 is modified to:

$$\begin{aligned}\boldsymbol{y}_1 &= \boldsymbol{W}_1\boldsymbol{\alpha}_1 + \boldsymbol{X}_1\boldsymbol{\beta_1} + \boldsymbol{\epsilon}_1 \\ \boldsymbol{y}_2 &= \boldsymbol{W}_2\boldsymbol{\alpha}_2 + \boldsymbol{X}_2\boldsymbol{\beta_2} + \boldsymbol{\epsilon}_2\end{aligned} \tag{7}$$

Here $\boldsymbol{W}_1$ is a $N_1 \times C_1$ matrix of covariates while $\boldsymbol{\alpha}_1$ denotes the fixed effect effect. Similarly, $\boldsymbol{W}_2$ is a $N_1 \times C_2$ matrix of covariates while $\boldsymbol{\alpha}_2$ is a fix effect effect of C_2-vector. We multiply each of the equations in Equation 7 by the projection matrices $\boldsymbol{V}_1 = \boldsymbol{I}_{N1} - \boldsymbol{W}_1(\boldsymbol{W}_1^T\boldsymbol{W}_1)^{-1}\boldsymbol{W}_1^T$ and $\boldsymbol{V}_2 = \boldsymbol{I}_{N2} - \boldsymbol{W}_2(\boldsymbol{W}_2^T\boldsymbol{W}_2)^{-1}\boldsymbol{W}_2^T$:

$$\begin{aligned}\boldsymbol{V}_1\boldsymbol{y}_1 &= \boldsymbol{V}_1\boldsymbol{X}_1\boldsymbol{\beta}_1 + \boldsymbol{V}_1\boldsymbol{\epsilon}_1 \\ \boldsymbol{V}_2\boldsymbol{y}_2 &= \boldsymbol{V}_2\boldsymbol{X}_2\boldsymbol{\beta}_2 + \boldsymbol{V}_2\boldsymbol{\epsilon}_2\end{aligned} \tag{8}$$

Similar to Equation 5, the MoM estimator is obtained by minimizing the sum of squared differences between the population and empirical covariance as :

$$\begin{aligned}&(\widehat{\gamma_g}, \widehat{\gamma_e}, \widehat{\sigma^2_{g1}}, \widehat{\sigma^2_{g2}}, \widehat{\sigma^2_{e1}}, \widehat{\sigma^2_{e2}}) = \\ &argmin_{\gamma_g,\gamma_e,\sigma^2_{g1},\sigma^2_{g2},\sigma^2_{e1},\sigma^2_{e2}} ||\widetilde{\boldsymbol{y}}\widetilde{\boldsymbol{y}}^T - (\begin{bmatrix}\sigma^2_{g1}\widetilde{\boldsymbol{K}_1} & \gamma_g\widetilde{\boldsymbol{K}_A} \\ \gamma_g\widetilde{\boldsymbol{K}_A}^T & \sigma^2_{g2}\widetilde{\boldsymbol{K}_2}\end{bmatrix} + \begin{bmatrix}\sigma^2_{e1}\boldsymbol{V}_1 & \gamma_e\boldsymbol{V}_1\boldsymbol{C}\boldsymbol{V}_2 \\ \gamma_e\boldsymbol{V}_2\boldsymbol{C}^T\boldsymbol{V}_1 & \sigma^2_{e2}\boldsymbol{V}_2\end{bmatrix})||^2_F\end{aligned}$$

where $\widetilde{\boldsymbol{y}} = \begin{bmatrix}\boldsymbol{V}_1\boldsymbol{y}_1 \\ \boldsymbol{V}_2\boldsymbol{y}_2\end{bmatrix}$, $\widetilde{\boldsymbol{K}_1} = \frac{\boldsymbol{V}_1\boldsymbol{X}_1\boldsymbol{X}_1^T\boldsymbol{V}_1}{M}$ and $\widetilde{\boldsymbol{K}_2} = \frac{\boldsymbol{V}_2\boldsymbol{X}_2\boldsymbol{X}_2^T\boldsymbol{V}_2}{M}$, $\widetilde{\boldsymbol{K}_A} = \frac{\boldsymbol{V}_1\boldsymbol{X}_1\boldsymbol{X}_2^T\boldsymbol{V}_2}{M}$, and $\widetilde{\boldsymbol{K}_C} = \frac{\boldsymbol{V}_1\boldsymbol{X}_1\boldsymbol{X}_2^T\boldsymbol{V}_2C^T}{M}$.

Thus the MoM estimator for genetic covariance satisfies the normal equations:

$$\begin{bmatrix}tr(\widetilde{\boldsymbol{K}_A}\widetilde{\boldsymbol{K}_A}^{\mathrm{T}}) & tr(\widetilde{\boldsymbol{K}_C}) \\ tr(\widetilde{\boldsymbol{K}_C}) & N\end{bmatrix}\begin{bmatrix}\widehat{\gamma_g} \\ \widehat{\gamma_e}\end{bmatrix} = \begin{bmatrix}\boldsymbol{y}_1^T\widetilde{\boldsymbol{K}_A}\boldsymbol{y}_2 \\ \boldsymbol{y}_1^T\boldsymbol{V}_1\boldsymbol{C}\boldsymbol{V}_2\boldsymbol{y}_2\end{bmatrix} \tag{9}$$

SCORE replaces $tr(\widetilde{\boldsymbol{K}_A}\widetilde{\boldsymbol{K}_A}^{\mathrm{T}})$ with an unbiased randomized estimate $\widetilde{L_B}$ using B random vec-

tors, $\boldsymbol{z}_1, \ldots, \boldsymbol{z}_B$, $z_b \in \mathbb{R}^{N_2}, b \in 1 \ldots B$ drawn independently from a distribution with zero mean and identity covariance matrix $\boldsymbol{I}_{N_2}$. The estimator of $tr(\widetilde{\boldsymbol{K}_A}\widetilde{\boldsymbol{K}_A}^{\mathrm{T}})$ is given by:

$$\widetilde{L_B} = \frac{1}{B}\frac{1}{M^2}\sum_b ||\boldsymbol{V}_1\boldsymbol{X}_1\boldsymbol{X}_2^T\boldsymbol{V}_2 z_b||_2^2$$

The SCORE estimator is thus obtained by solving Equation 9 by replacing $tr(\widetilde{\boldsymbol{K}_A}\widetilde{\boldsymbol{K}_A}^{\mathrm{T}})$ with $\widetilde{L_B}$. $\widetilde{L_B}$ is an unbiased estimator for $tr(\widetilde{\boldsymbol{K}_A}\widetilde{\boldsymbol{K}_A}^{\mathrm{T}})$ since

$$\begin{aligned}
\mathbb{E}[\widetilde{L_B}] &= \frac{1}{B}\frac{1}{M^2}\sum_b \mathbb{E}[\boldsymbol{z}_b^T(\boldsymbol{V}_1\boldsymbol{X}_1\boldsymbol{X}_2^T\boldsymbol{V}_2)^T\boldsymbol{V}_1\boldsymbol{X}_1\boldsymbol{X}_2^T\boldsymbol{V}_2\boldsymbol{z}_b] \\
&= \frac{1}{B}\sum_b \mathbb{E}[z_b^T\widetilde{\boldsymbol{K}_A}^T\widetilde{\boldsymbol{K}_A}\boldsymbol{z}_b] \\
&= \frac{1}{B}\sum_b \mathbb{E}[tr(z_b^T\widetilde{\boldsymbol{K}_A}^T\widetilde{\boldsymbol{K}_A}\boldsymbol{z}_b)] \\
&= \frac{1}{B}\sum_b \mathbb{E}[tr(\boldsymbol{z}_b\boldsymbol{z}_b^T\widetilde{\boldsymbol{K}_A}^T\widetilde{\boldsymbol{K}_A})] \\
&= \frac{1}{B}\sum_b tr(\mathbb{E}[\boldsymbol{z}_b\boldsymbol{z}_b^T\widetilde{\boldsymbol{K}_A}^T\widetilde{\boldsymbol{K}_A}]) \\
&= \frac{1}{B}\sum_b tr(\mathbb{E}[\boldsymbol{z}_b\boldsymbol{z}_b^T]\widetilde{\boldsymbol{K}_A}^T\widetilde{\boldsymbol{K}_A}) \\
&= \frac{1}{B}\sum_b tr(\boldsymbol{I}_{N2}\widetilde{\boldsymbol{K}_A}^T\widetilde{\boldsymbol{K}_A}) \\
&= tr(\widetilde{\boldsymbol{K}_A}^T\widetilde{\boldsymbol{K}_A})
\end{aligned}$$

The projection matrix $\boldsymbol{V}_1, \boldsymbol{V}_2$ need not need be computed explicitly. While computing $\boldsymbol{X}_2^T\boldsymbol{V}_2\boldsymbol{z}b$, we only need to compute the residual of $\boldsymbol{W}_1(\boldsymbol{W}_1^T\boldsymbol{W}_1)^{-1}\boldsymbol{W}_1{}^T\boldsymbol{z}_b$, where the additional computation has the complexity of $\mathcal{O}(N_1C_1)$ where C_1 is the number of covariates, which usually is a relatively small number.

Jackknife Standard Error

In order to compute the standard error using block Jackknife [1], we partition the standardized $N \times M$ genotype matrix $\boldsymbol{X}$ into J non-overlapping blocks, $\boldsymbol{X}^{(1)}, \boldsymbol{X}^{(2)}, \ldots, \boldsymbol{X}^{(J)}$ where $\boldsymbol{X}^{(j)} \in \{1, \ldots, J\}$ is an $N \times \frac{M}{J}$ matrix.

We define $\widehat{\rho_{g(j)}}$ to be the estimator of genetic correlation computed after excluding genotype block $\boldsymbol{X}^{(j)}$ from $\boldsymbol{X}$. Also, we define $\overline{\rho_{g(j)}} \equiv \frac{1}{J} \sum_j \widehat{\rho_{g(j)}}$

Thus, the jackknife estimate of the standard error is given as

$$\widehat{SE}(\widehat{\rho_g}) = \left[\frac{J-1}{J} \sum_{j=1}^{J} (\widehat{\rho_{g(j)}} - \overline{\rho_{g(j)}})^2 \right]^{\frac{1}{2}} \tag{10}$$

The computation of all of the $\widehat{\rho_{g(j)}}$ can be done with no additional computational cost relative to $\widehat{\rho_g}$.

References

1. Kunsch, H. R. (1989). The jackknife and the bootstrap for general stationary observations. *The Annals of Statistics*, **17**(3), 1217–1241.

2. Liberty, E. and Zucker, S. W. (2009). The mailman algorithm: A note on matrix–vector multiplication. *Information Processing Letters*, **109**(3), 179–182.
